# Unrecognized liver cirrhosis is common and associated with worse survival in hepatocellular carcinoma: A nationwide cohort study of 3473 patients

**DOI:** 10.1111/joim.13570

**Published:** 2022-10-03

**Authors:** Juan Vaz, Ulf Strömberg, Patrik Midlöv, Berne Eriksson, David Buchebner, Hannes Hagström

**Affiliations:** ^1^ Department of Clinical Sciences in Malmö, Center for Primary Health Care Research Lund University Malmö Sweden; ^2^ Department of Internal Medicine Halland Hospital Halmstad Halmstad Sweden; ^3^ Department of Research and Development Region Halland Halmstad Sweden; ^4^ Institute of Medicine, Sahlgrenska Academy University of Gothenburg Gothenburg Sweden; ^5^ Krefting Research Center, Institute of Medicine University of Gothenburg Gothenburg Sweden; ^6^ Department of Medicine, Huddinge Karolinska Institutet Stockholm Sweden; ^7^ Division of Hepatology, Department of Upper GI Diseases Karolinska University Hospital Stockholm Sweden; ^8^ Clinical Epidemiology Unit, Department of Medicine Karolinska Institutet Stockholm Sweden

**Keywords:** cirrhosis, comorbidity, etiology, hepatocellular carcinoma, prognosis, surveillance

## Abstract

**Background:**

Data on unrecognized liver cirrhosis in patients with hepatocellular carcinoma (HCC) are derived mainly from cohorts with a risk of selection bias.

**Objectives:**

In a population‐based cohort study we aimed to determine the proportion, characteristics, and prognosis of HCC in patients with unrecognized cirrhosis.

**Methods:**

Using the Swedish quality register for liver cancer and other nationwide registers, we identified all adults with HCC in Sweden between 2012 and 2018 (n = 3,473).

**Results:**

The final study cohort comprised 2670 patients with established cirrhosis, of which 1033 (39%) had unrecognized cirrhosis at HCC diagnosis. These patients were more often male, older, and had larger tumors, multinodular cancer, portal vein thrombosis, and extrahepatic metastasis compared to patients with known cirrhosis with HCC and under surveillance (34%). Compared to surveilled patients, those with unrecognized cirrhosis had worse median survival (0.89 years, 95% confidence interval [CI] = 0.78‐1.01 vs. 3.79 years, 95%CI = 3.19‐4.39), and an adjusted hazard ratio of 2.36 (95%CI = 2.09‐2.66). Patients with cirrhosis but not under surveillance (27%) and patients with unrecognized cirrhosis had similar characteristics, such as equal proportions diagnosed at late stage (79%).

**Conclusions:**

Cirrhosis is often not recognized in patients with HCC. Unrecognized cirrhosis is associated with more advanced HCC at diagnosis and a worse prognosis. More efforts are needed to diagnose cirrhosis at an earlier stage.

## Graphical abstract

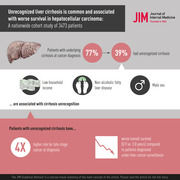

## Introduction

Primary liver cancer is the third leading cause of cancer‐related mortality worldwide [1]. Hepatocellular carcinoma (HCC) accounts for 75% to 85% of all primary liver cancer cases [1]. Liver cirrhosis is the main risk factor for HCC, and up to 90% of patients diagnosed with HCC have underlying cirrhosis [2]. Most international clinical guidelines advise HCC surveillance with biannual ultrasound in patients with cirrhosis [3, 4]. HCC surveillance has been associated with improved early detection, receipt of curative treatment, and overall survival in patients with cirrhosis [5]. However, a recent case‐control study did not show an improvement in mortality [6]. Indeed, the benefit and cost‐effectiveness of surveillance in cirrhosis are generally the subjects of extensive research and open controversy [7]. Objective assessment of the effect of surveillance on the quality of life and survival in patients with liver disease is challenging [7]. Cirrhosis is a silent disease that could go unnoticed for many years, and most patients are first diagnosed after the development of decompensation and organ dysfunction [8]. Delayed cirrhosis diagnosis is of particular concern because the cost‐effectiveness of HCC surveillance strategies depends on the likelihood of curative treatment receipt, which is limited in patients with decompensated cirrhosis [3, 4].

Health inequity plays an important role in cirrhosis and HCC [9]. Liver illness—especially cirrhosis—often carries important social stigma, leading to discrimination and reduced health‐seeking behaviors [9]. Surveillance strategies are also underused, with only <25% of patients with cirrhosis receiving consistent HCC surveillance [10]. Patients with low socioeconomic status or belonging to ethnic minorities may have lower surveillance receipt [11].

Previous studies from the United States indicate that cirrhosis might go unrecognized in 22% to 50% of patients diagnosed with HCC [12, 13, 14, 15, 16]. These results, however, are based on data from a limited number of patients, or the data could be prone to selection bias. The near completeness of data from Swedish registers infers a very low risk for selection bias [17]. Furthermore, contemporary studies examining the burden of unrecognized cirrhosis in patients diagnosed with HCC in Europe are limited.

The aims of this study were to i) determine the extent of unrecognized cirrhosis in patients with HCC in Sweden; ii) describe and compare patient characteristics in three subgroups: those diagnosed with HCC while under surveillance, those with known cirrhosis but who did not receive surveillance, and those with previously unrecognized cirrhosis; and iii) compare survival after HCC diagnosis between subgroups.

## Methods

### Study population

The Swedish quality register for cancers found in the liver, gallbladder, and bile ducts (SweLiv) was established in 2008. Since its creation, the register contains patient data from >95% of all known cases of HCC in Sweden (available at https://statistik.incanet.se/SweLiv/) [18]. The SweLiv, validated in 2014, consists of four modules containing clinical data, such as tumor characteristics, cirrhosis status, etiology, and treatment recommendations [18].

The first national treatment program for patients with HCC was launched in 2012 and has been updated regularly [18]. HCC surveillance strategies are described in this treatment program, which otherwise seeks to minimize health care inequities among patients diagnosed with HCC in Sweden.

All patients ≥18 years old registered in the SweLiv with a diagnosis of HCC (International Classification of Diseases 10^th^ Revision [ICD‐10] code C22.0) between 1 January 2012 and 31 December 2018 were included. The start of the study period considers the date of launching the national HCC surveillance guidelines. The end of the study period considers the standard delay of register updates in Sweden (1.5 years in some cases) [19] and guarantees a minimal follow‐up of 24 months for surviving patients diagnosed in 2018.

The unique Swedish personal identification number was used for data linkage between the SweLiv and other nationwide registers [20]. Sociodemographic data were retrieved from the Swedish Longitudinal Integrated Database for Health Insurance and Labor Market Studies maintained and monitored by Statistics Sweden [21]. Statistics Sweden is the Swedish government agency operating under the Ministry of Finance. A patient was considered to have a Nordic origin if born in Sweden, Norway, Denmark, Finland, or Iceland, and a non‐Nordic origin otherwise.

Household income (disposable income per household per consumption unit) was chosen as the indicator of individual‐level socioeconomic status [22]. Other individual‐level indicators, such as occupational social class or education, are more often associated with missing data for immigrants, which is not the case with household income [22]. Each patient was categorized as having a low, medium, or high household income based on the distribution of household income across all households in Sweden (Table S1) [22].

In addition to the SweLiv, we used data from the National Patient Register (NPR), the Prescribed Drug Register, and the Cause of Death Register to further define exposure and outcomes [23]. The NPR consists of ICD‐based diagnoses from inpatient and outpatient (since 2001) care in Sweden but does not capture primary care data [23]. Surgical procedures—including endoscopy, laparoscopy, and liver surgery—are also reported to the NPR [23]. The Prescribed Drug Register has data from prescribed drugs dispensed at any Swedish pharmacy since July 2005. The Cause of Death Register (currently >99% complete) uses ICD‐based codes to classify underlying and contributing causes of death. A detailed description of these registers is available elsewhere and in Figure S1 [24].

### Definition of cirrhosis, etiology, severity, number of visits, and comorbidities

A patient was regarded as having cirrhosis at the time of HCC diagnosis if i) registered as cirrhosis in the SweLiv or ii) at least one ICD‐10 cirrhosis‐related code was registered in the NPR between 1997 and 180 days after the date of HCC diagnosis (Table S1 and Fig. S2).

To further increase the sensitivity of our definition of cirrhosis, we additionally defined cirrhosis as present in patients with liver disease‐related ICD‐10 codes in the NPR if there were a dispensation for one of the following drugs: non‐selective beta‐blockers (propranolol or carvedilol), spironolactone, lactulose, or rifaximin.

Unrecognized cirrhosis was defined as patients without an ICD‐10 cirrhosis‐related code registered in the NPR between 1997 and 30 days before the date of HCC diagnosis but who were defined as having cirrhosis in the SweLiv. Patients registered as non‐cirrhotic in the SweLiv but with at least one ICD‐10 cirrhosis‐related code recorded in the NPR between 30 days before and 180 days after HCC diagnosis were also regarded as having unrecognized cirrhosis. The remaining patients with cirrhosis were considered to have recognized cirrhosis and were further divided into surveilled and non‐surveilled groups (see next section below). Patients not registered as having cirrhosis in the SweLiv or the NPR were excluded from the study.

The etiologies of cirrhosis were retrieved from the SweLiv or established by ICD‐10 codes in the NPR or the Cause of Death Register or by American Therapeutical Chemical (ATC) classification codes in the Prescribed Drug Register, as described in the supporting material (Table S1). Laboratory data were retrieved from the SweLiv. Decompensated cirrhosis was defined in patients with ascites or encephalopathy, jaundice (bilirubin ≥52μmol/L), or hypoalbuminemia (<28g/L).

Comorbidities (arterial hypertension, type 2 diabetes [T2D], coronary artery disease) were defined by ICD‐10 or ATC codes (Table S1). We also recorded the total number of visits registered in the NPR within 365 days before HCC diagnosis to identify potential opportunities for future screening initiatives of unrecognized cirrhosis.

### Diagnostic pathways, staging, and treatment receipt

Diagnostic pathways were retrieved from the SweLiv. Upon registration in the SweLiv, a diagnostic pathway is reported for each patient. A patient can be reported as being diagnosed under surveillance, clinically (diagnosis by intent ‐ symptomatic patient), or incidentally (*en passant* diagnosis by radiology or surgery). Clinically and incidentally diagnosed patients were considered non‐surveilled. A patient with unrecognized cirrhosis was also regarded as non‐surveilled. Hence, three subgroups were defined: patients with known cirrhosis diagnosed with HCC while under surveillance, patients with known cirrhosis diagnosed without receiving surveillance, and patients with unrecognized cirrhosis.

Data on surveillance did not include the date of inclusion into a surveillance program, radiological method of choice, time interval between surveillances, or rate of completeness. Moreover, it was impossible to determine whether patients with known cirrhosis diagnosed without receiving surveillance had previously been excepted from surveillance, or if they were diagnosed with HCC between surveillances.

HCC stage at diagnosis was defined according to the Swedish treatment algorithm for HCC (Fig. S3), an adaptation of the Barcelona Clinic Liver Cancer (BCLC) staging system [25]. Early‐stage HCC was defined as BCLC 0‐A, and late‐stage as BCLC B‐D. BCLC stage was chosen as a proxy for other clinically important patient‐ and tumor‐associated variables. The BCLC stage variable had a high level of completeness (98%), which was not the case for other individual‐level tumor‐related variables or performance status (Table S1) [22].

Treatment recommendations were retrieved from the SweLiv and classified as i) curative intent (transplantation, resection, or ablation), ii) palliative (transarterial chemoembolization, or systemic chemotherapy), iii) and best supportive care (Table S1). Treatments with curative intent were verified by cross‐linkage to surgical data registered in the NPR. Patients with multiple treatments with curative intent reported transplantation as the primary treatment, regardless of previous surgery; otherwise, resection was considered the treatment of choice if performed before ablation, and ablation if performed before resection [18].

### Outcomes

Survival time was defined as the time from HCC diagnosis to date of death, the latter being retrieved from the Cause of Death Register. Each patient was followed until date of death or emigration or the end of the study period (31 December 2020), whichever occurred first.

### Statistical analysis

Categorical and dichotomous variables were presented as numbers and percentages, and continuous data as medians and interquartile ranges (IQRs). Differences between groups were tested using the chi‐square test (categorical variables) or the Mann‐Whitney U test (continuous variables). Logistic regression was used to identify patient characteristics associated with the i) likelihood of belonging to a specific patient group compared to the other two groups or compared to the aggregate of the other two groups and ii) likelihood of late‐stage HCC at diagnosis.

Multivariable logistic regression models were constructed using clinically relevant variables. The adjusted models included sex, age (continuous variable), country of birth, household income, etiology, number of visits (continuous variable), arterial hypertension, T2D, coronary artery disease, and decompensation. Variables with a p‐value ≤0.20 were included in the adjusted models. Model fit was assessed using the Hosmer‐Lemeshow statistic [26].

Kaplan‐Meier estimates with Greenwood confidence intervals (CIs) were used to determine median survival and survival probabilities between the three subgroups. Survival curves were compared using the log‐rank test. Unadjusted and adjusted hazard ratios (HRs and aHRs) were estimated by employing Cox regression modeling. T2D (yes/no) was incorporated into the Cox models based on prior clinical knowledge [27], whereas the other variables were included based on a p‐value ≤0.20.

The final Cox model was adjusted for age (continuous variable), country of birth, household income, cirrhosis recognition status, etiology of cirrhosis, arterial hypertension, T2D, coronary artery disease, and decompensation. The assumption of proportional hazards was evaluated using the scaled Schoenfeld residuals [28].

Missing data were presented as numbers and percentages. All data from a patient with missing data were omitted from multivariable analyses. A p‐value <0.05 (two‐tailed) was considered statistically significant. All tests were conducted using Stata v.17 (StataCorp, College Station, TX, USA).

The study was approved by the Central Ethical Review Board in Sweden (Decision Number 2020–04430). Due to the retrospective nature of the study, patient consent was not required.

## Results

**Fig. 1:**
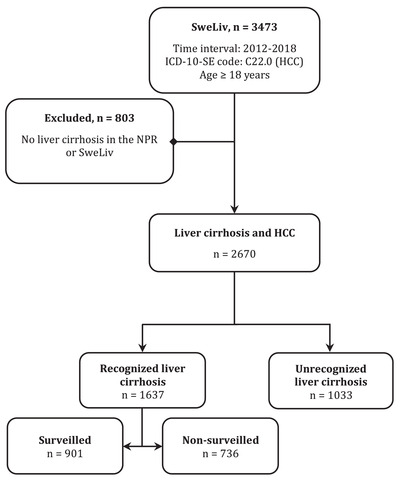
Patients diagnosed with hepatocellular carcinoma (HCC) in Sweden between 1 January 2012 and 31 December 2018 and registered in the Swedish quality register for cancers found in the liver, gallbladder, and bile ducts (SweLiv) or the National Patient Register (NPR) as having liver cirrhosis at the time of HCC diagnosis. Identification flowchart. ICD‐10‐SE: International Classification of Diseases 10^th^ Revision ‐ Swedish Edition

Some 3473 cases with HCC were included (Fig. 1). Of these, 2670 patients (77%) were judged to have cirrhosis at the time of HCC diagnosis. Unrecognized cirrhosis was found in 1033 patients (30% of overall and 39% of all cirrhosis). No patient with unrecognized cirrhosis had a prior diagnosis of liver disease registered in the NPR until a month before HCC diagnosis. In total, 901 patients were diagnosed with HCC while under surveillance (Table 1). These patients accounted for 26, 34, and 55% of i) all patients diagnosed with HCC, ii) all patients with underlying cirrhosis, and iii) patients with previously recognized cirrhosis, respectively.

**Table 1:** Baseline characteristics of patients with cirrhosis diagnosed with HCC in Sweden between 2012 and 2018

	Recognized			
	Surveillance	No surveillance	Unrecognized	Total
Total, *n* (%)	901 (34)	736 (27)	1033 (39)	2670 (100)
Sex (male)	684 (76)	543 (74)	855 (83)	2082 (78)
Median age (years)	65 (59‐71)	68 (61‐74)	69 (62‐76)	67 (61‐74)
Country of birth				
Nordic	740 (82)	636 (86)	867 (84)	2243 (84)
Non‐Nordic	161 (18)	100 (14)	166 (16)	427 (16)
Household income				
High	140 (16)	99 (14)	122 (12)	361 (14)
Medium	379 (42)	268 (36)	406 (39)	1053 (39)
Low	382 (42)	369 (50)	505 (49)	1256 (47)
Etiology				
Viral hepatitis	233 (26)	117 (16)	200 (20)	550 (21)
Viral hepatitis + ALD	258 (29)	167 (22)	161 (15)	586 (22)
ALD	176 (19)	196 (27)	223 (22)	595 (22)
NAFLD	87 (10)	108 (15)	225 (22)	420 (16)
Other	105 (12)	111 (15)	63 (6)	279 (10)
Cryptogenic	42 (4)	37 (5)	161 (15)	240 (9)
Decompensation^a^	225 (25)	417 (57)	374 (36)	1016 (38)
Unknown	54 (6)	59 (8)	117 (1)	230 (9)
Comorbidity				
Arterial hypertension	484 (54)	421 (57)	585 (57)	1490 (56)
Type 2 diabetes	338 (38)	331 (45)	421 (41)	1090 (41)
CAD	93 (10)	130 (18)	184 (18)	407 (15)
N of visits in NPR^b^	3 (2‐7)	4 (2‐7)	2 (1‐4)	3 (1‐6)
Diagnostic pathway				
Surveillance	901 (100)	0	0	901 (34)
Clinical	0	582 (79)	815 (79)	1397 (52)
Incidental	0	154 (21)	218 (21)	372 (14)
Ascites				
Absent	690 (77)	378 (51)	671 (65)	1739 (65)
Slight	102 (11)	115 (16)	115 (11)	332 (12)
Moderate	50 (5)	163 (22)	108 (11)	321 (12)
Unknown	59 (7)	80 (11)	139 (13)	278 (11)
Encephalopathy				
Absent	811 (90)	545 (74)	818 (79)	2174 (81)
Grade 1–2	20 (2)	45 (6)	14 (1)	79 (3)
Grade 3–4	3 (≪1)	14 (2)	7 (<1)	24 (1)
Unknown	67 (7)	132 (18)	194 (19)	393 (15)
Albumin (g/L)				
Median	34 (30‐38)	29 (25‐33)	31 (26‐36)	32 (27‐36)
Unknown	152 (17)	135 (18)	225 (22)	512 (19)
Bilirubin (μmol/L)				
Median	16 (10‐26)	22 (13‐42)	15 (10‐27)	17 (11‐31)
Unknown	147 (16)	120 (16)	193 (19)	460 (17)
PT (INR)				
Median	1.2 (1.1‐1.3)	1.3 (1.1‐1.4)	1.2 (1.1‐1.3)	1.2 (1.1‐1.4)
Unknown	151 (17)	134 (18)	219 (21)	504 (19)
Performance status				
(ECOG)				
0	484 (54)	191 (26)	261 (25)	936 (35)
1	239 (27)	159 (22)	236 (23)	634 (24)
≥2	101 (11)	259 (35)	348 (34)	708 (26)
Unknown	77 (8)	127 (17)	188 (18)	392 (15)
AFP (μg/L)				
Median	10 (5‐64)	22 (6‐853)	37 (6‐1128)	17 (5‐405)
Unknown	260 (29)	243 (33)	321 (31)	824 (31)
Tumor size (mm)				
Median	29 (20‐42)	40 (25‐65)	55 (34‐95)	40 (25‐70)
<20	266 (30)	119 (16)	109 (11)	494 (18)
20‐29	247 (27)	122 (17)	102 (10)	471 (18)
≥30	354 (39)	400 (54)	707 (68)	1461 (55)
Unknown	34 (4)	95 (13)	115 (11)	244 (9)
Number of tumors				
1	483 (54)	310 (42)	418 (41)	1211 (46)
2‐3	279 (31)	197 (27)	229 (22)	705 (26)
>3	92 (10)	96 (13)	185 (18)	373 (14)
Unknown	47 (5)	133 (18)	201 (19)	381 (14)
Metastasis				
Regional	49 (5)	78 (11)	180 (17)	307 (11)
Extrahepatic	37 (4)	112 (15)	225 (22)	374 (14)
PVT	82 (9)	174 (24)	293 (28)	549 (21)
BCLC stage				
0‐A	456 (51)	144 (20)	195 (19)	795 (30)
B‐D	414 (46)	583 (79)	821 (79)	1818 (68)
Missing	31 (3)	9 (1)	17 (2)	57 (2)
Treatment				
Transplantation	137 (15)	54 (7)	34 (3)	225 (9)
Resection	158 (18)	47 (6)	117 (11)	322 (12)
Ablation	276 (31)	87 (12)	93 (9)	456 (17)
Palliative	169 (18)	145 (20)	258 (25)	572 (21)
BSC	161 (18)	403 (55)	531 (52)	1095 (41)

AFP: Alpha‐fetoprotein; ALD: Alcoholic liver disease; BCLC: Barcelona Clinic Liver Cancer staging system; BSC: Best supportive care; CAD: Coronary artery disease; ECOG: Eastern Cooperative Oncology Group; HCC: Hepatocellular carcinoma; NAFLD: Non‐alcoholic fatty liver disease; NPR: National Patient Registry; PT (INR): Prothrombin time (international normalized ratio); PVT: Portal vein thrombosis. Nordic country of birth: Sweden, Denmark, Finland, Iceland, and Norway. Household income is defined as disposable income per household per consumption unit. Median age, tumor size, and laboratory values presented with corresponding interquartile range in parenthesis. ^a^Decompensated cirrhosis is defined as ascites or encephalopathy or bilirubin≥52 μmol/L or albumin<28 g/L. ^b^Registered within 365 days before HCC diagnosis

The median age was 67 years, and most patients were men (78%) (Table 1). Nearly half of the patients had a low household income (47%). Viral hepatitis and alcoholic liver disease (ALD)—alone or combined—were the principal causes of HCC, followed by non‐alcoholic fatty liver disease (NAFLD). Overall, 1003 patients (38%) received treatment with curative intention.

Subgroups, where HCC was not diagnosed during surveillance, shared several patient characteristics independently of whether cirrhosis was known before HCC diagnosis (Table 1 and Table S2). Although patients with unrecognized cirrhosis had more multinodular tumors (18 vs. 13%, p = 0.006) and extrahepatic metastasis (22 vs. 15%, p<0.001) compared to patients with known cirrhosis diagnosed without receiving surveillance, both subgroups had the same proportion of patients diagnosed with a BCLC B‐D HCC (79%, p = 0.905) and received curative treatment to a similar extent (23 vs. 25%, p = 0.496). Decompensation was more common in patients diagnosed with known cirrhosis without surveillance (57 vs. 36%, p<0.001).

Patients with unrecognized cirrhosis had a median of two visits (IQR 1–4) registered in the NPR within 365 days before HCC diagnosis. Of these patients, 298 (29%) had no visits registered before HCC diagnosis. The remaining 735 patients (71%) had a combined total of 3073 visits registered, of which 75% were outpatient visits. Most visits were registered at internal medicine clinics (32%), followed by surgical (30%) and psychiatric clinics (10%) (Fig. S4).

Patients diagnosed under surveillance had distinct differences compared to the subgroups in which HCC was not diagnosed during surveillance (Table S3). Patients diagnosed under surveillance were younger (65 vs. 69 years, p<0.001), more frequently had viral hepatitis (26 vs. 18%, p<0.001), and had ALD (19 vs. 24%, p = 0.016), NAFLD (10 vs. 19%, p<0.001), T2D (38 vs. 43%, p = 0.014), and coronary artery disease (10 vs. 18%, p<0.001) to a lesser extent. These patients also had better performance status, smaller tumors (median 29 mm vs. 50 mm, p<0.001), less decompensation (25 vs. 45%, p<0.001), and had multinodular cancer (10 vs. 16%, p<0.001), portal vein thrombosis (9 vs. 26%, p<0.001) and extrahepatic metastasis to a lesser extent (4 vs. 19%, p<0.001). Finally, 51% of patients under surveillance were diagnosed with a BCLC 0‐A HCC.

### Associations between patient groups

Logistic regression models were performed to ascertain the effect of different variables on the likelihood of belonging to a specific patient group. Compared to patients diagnosed under surveillance, patients with known cirrhosis diagnosed without surveillance were more likely to have a low household income (adjusted odds ratio [aOR] 1.47 95%CI = 1.04‐2.07), coronary artery disease (aOR 1.58 95%CI = 1.13‐2.22), and decompensated cirrhosis (aOR 4.27 95%CI = 3.40‐5.35). Patients with known cirrhosis diagnosed without surveillance also had a higher likelihood of non‐viral cirrhosis, with the highest aOR estimated for NAFLD (2.05, 95%CI = 1.31‐3.21) (Table S4).

Compared to patients diagnosed under surveillance, patients with unrecognized cirrhosis were more likely male (aHR 1.79 95%CI = 1.36‐2.35) and more likely to have a low household income (aOR 1.54 95%CI = 1.11‐2.14). Moreover, patients with unrecognized cirrhosis more likely had NAFLD (aOR 3.18 95%CI = 2.12‐4.77), cryptogenic cirrhosis (aOR 3.12 95%CI = 1.96‐4.96), decompensation (aOR 2.32 95%CI = 1.84‐2.92), and coronary artery disease (aOR 1.63 95%CI = 1.17‐2.27) (Table S4).

Compared to patients with known cirrhosis diagnosed without surveillance, patients with unrecognized cirrhosis were more likely male (aOR 1.83 95%CI = 1.38‐2.43), but no statistically significant associations between age, country of birth, household income, or comorbidity were observed. Patients with unrecognized cirrhosis had a higher likelihood of cryptogenic cirrhosis (aOR 1.87 95%CI = 1.12‐3.13), a similar probability of NAFLD, and were lower for most of the remaining etiologies (Table S5). Decompensated cirrhosis was less likely in patients with unrecognized cirrhosis (aOR 0.47 95%CI = 0.38‐0.59).

### Likelihood of unrecognized cirrhosis

Compared to patients with known cirrhosis, patients with unrecognized cirrhosis were more likely male (aOR 1.80 95%CI = 1.45‐2.25). They also had an increased likelihood of having a low household income (aOR 1.39 95%CI = 1.06‐1.83), NAFLD (aOR 1.94 95%CI = 1.45‐2.60), and cryptogenic cirrhosis (aOR 2.77 95%CI = 1.95‐3.93). The number of health care visits in the year preceding the HCC diagnosis was associated with a lower aOR (0.84 95%CI = 0.82‐0.87) for unrecognized cirrhosis (Table 2).

**Table 2:** Factors associated with the likelihood of having unrecognized cirrhosis before being diagnosed with HCC

	Univariable		Multivariable	
	OR (95% CI)	P‐value	aOR (95% CI)	P‐value
Sex				
Female	1.0 (ref)		1.0 (ref)	
Male	1.61 (1.32‐1.9)	<0.001	1.80 (1.45‐2.25)	<0.001
Age (years)			1.01 (1.00‐1.02)	0.022
Country of birth				
Nordic	1.0 (ref)			
Non‐Nordic	1.01 (0.82‐1.25)	0.931		
Household income				
High	1.0 (ref)		1.0 (ref)	
Medium	1.23 (0.96‐1.58)	0.107	1.16 (0.88‐1.52)	0.269
Low	1.32 (1.03‐1.68)	0.028	1.39 (1.06‐1.83)	0.017
Etiology				
Viral hepatitis	1.0 (ref)		1.0 (ref)	
Viral hepatitis + ALD	0.66 (0.52‐0.85)	0.001	0.70 (0.54‐0.92)	0.009
ALD	1.05 (0.82‐1.33)	0.696	0.95 (0.73‐1.23)	0.667
NAFLD	2.02 (1.56‐2.62)	<0.001	1.94 (1.45‐2.60)	<0.001
Other	0.51 (0.37‐0.71)	<0.001	0.56 (0.40‐0.81)	0.002
Cryptogenic	3.57 (2.59‐4.91)	<0.001	2.77 (1.95‐3.93)	<0.001
Decompensation^a^	0.95 (0.80‐1.12)	0.529		
N of visits NPR^b^	0.84 (0.81‐0.86)	<0.001	0.84 (0.82‐0.87)	<0.001
Comorbidity				
Arterial hypertension	1.06 (0.90‐1.23)	0.495		0.660
Type 2 diabetes	0.99 (0.85‐1.17)	0.954		0.465
CAD	1.37 (1.11‐1.70)	0.003	1.26 (0.99‐1.60)	0.064

ALD: Alcoholic liver disease; CAD: Coronary artery disease; CI: Confidence interval; HCC: Hepatocellular carcinoma; NAFLD: Non‐alcoholic fatty liver disease; NPR: National Patient Registry; OR: Odds ratio. Nordic country of birth: Sweden, Denmark, Finland, Iceland, and Norway. Household income is defined as disposable income per household per consumption unit. Results from univariable and multivariable logistic regression models. The multivariable model, which included all the variables in this table, was statistically significant compared to the null model (Chi‐square (11) = 422.336, p<0.001 and correctly classified 68% of the cases.

^a^ Decompensated cirrhosis is defined as ascites or encephalopathy or bilirubin≥52 μmol/L or albumin<28 g/L. ^b^Registered within 365 days before HCC diagnosis.

### Likelihood of being diagnosed outside of surveillance

Because patient characteristics in the subgroups in which HCC was not diagnosed during surveillance differed from those observed in patients diagnosed under surveillance, logistic regression models were performed to ascertain the effect of different variables on the likelihood of being diagnosed under non‐surveillance (Table S6).

Male sex (aOR 1.44 95%CI = 1.15‐1.81), low household income (aOR 1.57 95%CI = 1.19‐2.09), and coronary artery disease (aOR 1.55 95%CI = 1.17‐2.07) were associated with an increased likelihood of diagnosis under non‐surveillance compared to their corresponding reference categories. NAFLD (aOR 2.46 95%CI = 1.72‐3.53) and cryptogenic cirrhosis (aOR 2.75 95%CI = 1.78‐4.26) were associated with an increased likelihood of diagnosis under non‐surveillance compared to viral hepatitis. Cirrhosis decompensation was also associated with an increased probability of being diagnosed under non‐surveillance (aOR 2.98 95%CI = 2.46‐3.63).

### Likelihood of late‐stage HCC at diagnosis

We further examined the association between the study variables and the likelihood of late‐stage HCC diagnosis (BCLC 0‐A vs. B‐D) (Table 3). Compared to patients diagnosed under surveillance, the two subgroups in which HCC was not diagnosed during surveillance had an increased likelihood of late‐stage HCC in the multivariable model: aOR = 4.19 (95%CI = 3.32‐5.29) for patients with known cirrhosis diagnosed without surveillance and 3.96 (95%CI = 3.18‐4.94) for patients with unrecognized cirrhosis.

**Table 3:** Different factors and their association to the likelihood of late‐stage HCC diagnosis in Sweden (2012‐2018)

	Univariable		Multivariable	
	OR (95% CI)	p‐value	aOR (95% CI)	p‐value
Sex				
Female	1.0 (ref)	‐	1.0 (ref)	
Male	1.25 (1.03‐1.52)	0.026	1.36 (1.09‐1.71)	0.008
Age (years)	1.04 (1.03‐1.05)	<0.001	1.03 (1.02‐1.04)	<0.001
Country of birth				
Nordic	1.0 (ref)		1.0 (ref)	
Non‐Nordic	0.63 (0.50‐0.78)	<0.001	0.66 (0.51‐0.85)	0.002
Household income				
High	1.0 (ref)		1.0 (ref)	
Medium	1.38 (1.07‐1.77)	0.012	1.40 (1.07‐1.84)	0.015
Low	1.85 (1.44‐2.37)	<0.001	1.89 (1.43‐2.52)	<0.001
Liver cirrhosis				
Recognized (surveillance)	1.0 (ref)		1.0 (ref)	
Recognized (non‐surveillance)	4.46 (3.56‐5.59)	<0.001	4.19 (3.32‐5.29)	<0.001
Unrecognized	4.64 (3.78‐5.69)	<0.001	3.96 (3.18‐4.94)	<0.001
Etiology				
Viral hepatitis	1.0 (ref)		1.0 (ref)	
Viral hepatitis + ALD	1.14 (0.90‐1.46)	0.281	0.97 (0.73‐1.29)	0.856
ALD	1.44 (1.12‐1.85)	0.004	0.99 (0.74‐1.32)	0.923
NAFLD	1.69 (1.28‐2.24)	<0.001	0.90 (0.63‐1.28)	0.556
Other	1.50 (1.10‐2.06)	0.011	1.29 (0.93‐1.86)	0.176
Cryptogenic	3.54 (2.37‐5.30)	<0.001	1.97 (1.26‐3.01)	0.003
N of visits NPR^a^	0.98 (0.96‐0.99)	0.010	0.99 (0.98‐1.01)	0.595
Comorbidity				
Arterial hypertension	0.81 (0.68‐0.96)	0.013	0.61 (0.50‐0.75)	<0.001
Type 2 diabetes	1.07 (0.91‐1.27)	0.420	1.17 (0.94‐1.47)	0.157
CAD	1.72 (1.33‐2.22)	<0.001	1.32 (0.99‐1.74)	0.058

ALD: Alcoholic liver disease; CAD: Coronary artery disease; CI: Confidence interval; HCC: Hepatocellular carcinoma; NAFLD: Non‐alcoholic fatty liver disease; NPR: National Patient Registry; OR: Odds ratio. Nordic country of birth: Sweden, Denmark, Finland, Iceland, and Norway. Household income is defined as disposable income per household per consumption unit. Results from univariable and multivariable logistic regression models. HCC stage was classified according to the Barcelona Clinic Liver Cancer (BCLC) staging system as early‐stage (BCLC 0‐A) and late‐stage (BCLC B‐D).

The multivariable model included all shown variables, was statistically significant compared to the null model (Chi‐square (16) = 406.096, p<0.001) and correctly classified 73% of the cases.

^a^ Registered within 365 days before HCC diagnosis.

Being born outside a Nordic country (aOR = 0.66 95%CI = 0.51‐0.85) and arterial hypertension (aOR = 0.61 95%CI = 0.50‐0.75) were associated with a decreased likelihood of being diagnosed with HCC at a late stage compared to their corresponding reference categories. Male sex (aOR = 1.36 95%CI = 1.09‐1.71), medium (aOR = 1.40 95%CI = 1.07‐1.84) or low household income (aOR = 1.89 95%CI = 1.43‐2.52), and cryptogenic cirrhosis (aOR = 1.97 95%CI = 1.26‐3.01) were all associated with an increased likelihood of a late‐stage diagnosis compared to their corresponding reference counterparts.

### Survival analysis

During an accumulated follow‐up time of 5847 person‐years, 1974 patients (74%) died, with follow‐up censored for the remaining 696 patients. Median survival was 1.48 years (95%CI = 1.36‐1.60) and the 1‐ and 5‐year survival probabilities were 0.58 (95%CI = 0.56‐0.60) and 0.24 (95%CI = 0.22‐0.26), respectively (Table S7).

**Fig. 2:**
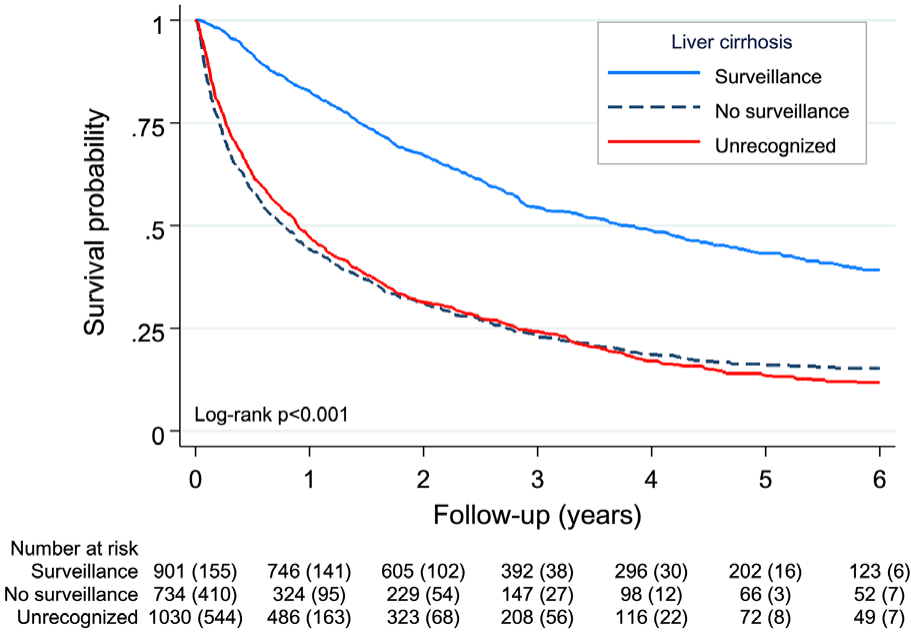
Kaplan‐Meier survival curves in a cohort of 2670 patients diagnosed with hepatocellular carcinoma (HCC) in Sweden (2012‐2018). Survival probabilities compared by liver cirrhosis status and diagnostic pathway: recognized and surveillance, recognized and no surveillance, and unrecognized liver cirrhosis. The number of terminal events is shown in parentheses. Time after HCC diagnosis was limited to 6 years

Patients diagnosed under surveillance had a median survival of 3.79 years (95%CI = 3.19‐4.39). In contrast, patients with known cirrhosis diagnosed without surveillance had a median survival of 0.76 years (95%CI = 0.63‐0.89) and patients with unrecognized cirrhosis 0.89 years (95%CI = 0.78‐1.01) (Fig. 2). Compared to patients diagnosed under surveillance, patients with known cirrhosis diagnosed without surveillance had a higher rate of mortality (aHR 2.18, 95%CI = 1.92‐2.48), similar to patients with unrecognized cirrhosis (aHR 2.36, 95%CI = 2.09‐2.66). After further adjustment for BCLC stage, the mortality rate was somewhat reduced for patients with known cirrhosis (aHR 1.89, 95% CI, 1.67‐2.14) and patients with unrecognized cirrhosis (aHR 1.76, 95% CI, 1.56‐1.98) (Table 4).

**Table 4:** Univariable and multivariable estimates for overall mortality in 2670 patients with cirrhosis diagnosed with HCC in Sweden between 2012 and 2018

	Univariable		Multivariable 1		Multivariable 2	
	HR (95% CI)	p‐value	aHR (95% CI)	p‐value	aHR (95% CI)	p‐value
Sex						
Female	1.0 (ref)					
Male	1.00 (0.90‐1.12)	0.972				
Age (years)	1.03 (1.02‐1.03)	<0.001	1.02 (1.01‐1.03)	<0.001	1.01 (1.00‐1.02)	<0.001
Country of birth						
Nordic	1.0 (ref)		1.0 (ref)		1.0 (ref)	
Non‐Nordic	0.77 (0.68‐0.87)	<0.001	0.79 (0.63‐0.84)	<0.001	0.89 (0.78‐1.03)	0.897
Household income						
High	1.0 (ref)		1.0 (ref)		1.0 (ref)	
Medium	1.29 (1.11‐1.50)	<0.001	1.13 (0.97‐1.32)	0.115	1.21 (1.04‐1.41)	0.013
Low	1.54 (1.34‐1.78)	<0.001	1.35 (1.16‐1.58)	<0.001	1.33 (1.14‐1.55)	<0.001
Cirrhosis						
Recognized (surveillance)	1.0 (ref)		1.0 (ref)		1.0 (ref)	
Recognized (non‐surveillance)	2.64 (2.34‐2.98)	<0.001	2.18 (1.92‐2.48)	<0.001	1.89 (1.67‐2.14)	<0.001
Unrecognized	2.59 (2.32‐2.90)	<0.001	2.36 (2.09‐2.66)	<0.001	1.76 (1.56‐1.98)	<0.001
Etiology						
Viral hepatitis	1.0 (ref)		1.0 (ref)		1.0 (ref)	
Viral hepatitis + ALD	1.06 (0.93‐1.23)	0.395	0.91 (0.78‐1.07)	0.254	0.94 (0.81‐1.10)	0.433
ALD	1.32 (1.15‐1.52)	<0.001	0.97 (0.83‐1.13)	0.687	1.06 (0.92‐1.24)	0.420
NAFLD	1.60 (1.38‐1.86)	<0.001	1.09 (0.90‐1.31)	0.389	1.25 (1.04‐1.50)	0.015
Other	1.25 (1.05‐1.48)	0.011	0.99 (0.82‐1.20)	0.954	0.97 (0.80‐1.16)	0.965
Cryptogenic	2.06 (1.74‐2.44)	<0.001	1.25 (1.02‐1.53)	0.030	1.21 (1.01‐1.47)	0.046
Decompensation^a^	2.38 (2.17‐2.61)	<0.001	2.33 (2.11‐2.57)	<0.001	Excluded	
Comorbidity						
Arterial hypertension	0.88 (0.80‐0.96)	0.005	0.75 (0.68‐0.83)	<0.001	0.78 (0.71‐0.86)	<0.001
Type 2 diabetes	1.05 (0.96‐1.14)	0.325	1.02 (0.91‐1.15)	0.756	0.99 (0.88‐1.11)	0.807
CAD	1.35 (1.20‐1.51)	<0.001	1.13 (0.99‐1.29)	0.069	1.02 (0.90‐1.16)	0.707
BCLC						
0‐A	1.0 (ref)		Excluded		1.0 (ref)	
B‐D	5.30 (4.68‐5.99)	<0.001	Excluded		4.23 (3.77‐4.87)	<0.001

ALD: Alcoholic liver disease; BCLC: Barcelona Clinic Liver Cancer; CAD: Coronary artery disease; CI: Confidence interval; HCC: Hepatocellular carcinoma; HR: Hazard ratio; NAFLD: Non‐alcoholic fatty liver disease. Nordic country of birth: Sweden, Denmark, Finland, Iceland, and Norway. Household income is defined as disposable income per household per consumption unit. Cox regression models were used to calculate HR and adjusted HR (aHR) for death. Each patient was followed‐up from the date of HCC diagnosis until the date of death, or until 31 December 2020, whichever occurred first. Multivariable models were adjusted for all variables in this table in the corresponding model. ^a^Decompensated cirrhosis is defined as ascites or encephalopathy or bilirubin≥52 μmol/L or albumin<28 g/L.

Some 205 patients (28%) in the group of patients with known cirrhosis diagnosed without surveillance had no evidence of cirrhosis decompensation and had a performance status of ≤1. In sensitivity analyses, these patients and patients diagnosed under surveillance were compared to patients with unrecognized cirrhosis. Even in these models, patients with unrecognized cirrhosis had markedly increased mortality for unadjusted (HR 2.53, 95%CI = 2.29‐2.81) and adjusted (aHR 2.15, 95%CI = 1.92‐2.41) HRs. After further adjustment for BCLC stage, the mortality for patients with unrecognized cirrhosis was again somewhat reduced compared to the main model (aHR = 1.75, 95%CI = 1.57‐1.96) (Table S8). Altogether, mortality estimates from the original models (Table 4) were comparable to those obtained from sensitivity analyses (Model 1: HR = 2.59 vs. 2.53; model 2: aHR = 2.36 vs. 2.15; and model 3: aHR = 1.76 vs. 1.75).

## Discussion

In this large national cohort study, we found that 4 of 10 patients diagnosed with HCC with underlying cirrhosis had their cirrhosis diagnosed only when HCC was detected. Patients with unknown cirrhosis had a four‐fold higher probability of presenting with late‐stage HCC at diagnosis than patients with known cirrhosis where HCC was diagnosed under surveillance. Specifically, they had larger tumor size, more multinodular tumors and portal vein thrombosis, and extrahepatic metastasis to a greater extent. Moreover, patients with unrecognized cirrhosis had considerably worse survival than patients diagnosed with HCC under surveillance, but they had comparable survival to patients with known cirrhosis where HCC was diagnosed outside of surveillance or where surveillance was never initiated. These findings—in line with several studies from the United States (12‐16)—emphasize the importance of early cirrhosis detection and the need for improved diagnostic pathways.

We identified several risk factors associated with unrecognized cirrhosis. Patients with unrecognized cirrhosis were more often male, had a low household income, and had a higher frequency of NAFLD‐related or cryptogenic cirrhosis. Most patients with unrecognized cirrhosis also had at least one NAFLD‐related comorbidity and a median of two visits recorded in the NPR within 365 days before HCC diagnosis. Data on missed opportunities for early diagnosis of end‐stage liver disease illustrate current health care challenges [9] but also provide information for the design of possible targeted interventions.

Compared to patients with viral hepatitis, NAFLD has been linked to an increased risk for unrecognized cirrhosis [14]. Here, NAFLD was the most common cause of HCC in patients with unrecognized cirrhosis. NAFLD‐HCC was also linked to worse survival compared to viral hepatitis or ALD alone or combined. This finding is of particular concern as NAFLD is the most common liver disease, with a worldwide prevalence of 25% [29]. Moreover, it has become the most common cause of HCC among Medicare users in the United States [30]. NAFLD is also a leading cause of cirrhosis in Sweden and an increasing indication for liver transplantation in the Nordic countries [31, 32].

The early detection of HCC in patients with NAFLD is difficult because patients developing HCC more often do not have underlying cirrhosis [33]. NAFLD cirrhosis, however, often goes unrecognized and is not seldom associated with HCC [34]. Our findings support previous observations and stress the need for greater awareness and better diagnostic methods for the early detection of cirrhosis, especially in patients with NAFLD.

Arterial hypertension and T2D were common in patients with unrecognized cirrhosis. The association between T2D and cirrhosis and HCC is well‐known [35]. Although clinical guidelines recommend using liver function tests for the early detection of NAFLD in patients with diabetes and vice versa [36], liver disease is an overlooked complication in patients with diabetes [37]. The awareness of NAFLD in patients with T2D in Swedish primary health care is low, and a previous study found that liver disease was seldom followed up [38].

Because most patients with arterial hypertension or diabetes in Sweden are followed up through primary care centers, the inclusion of non‐invasive tests in follow‐up guidelines for patients with arterial hypertension—especially for those with other risk factors for liver disease (e.g., impaired glucose tolerance and coronary artery disease)—might be of value for the early recognition of patients with cirrhosis. However, overdiagnosis due to poor specificity is a concern [39].

The high proportion of patients with unrecognized cirrhosis being in contact with specialized health care within 1 year before HCC diagnosis signals possible screening opportunities. However, it also highlights that possible cirrhosis is often overlooked and not actively investigated in at‐risk individuals.

The Swedish welfare system is characterized by its tax‐financed structure, which aims to offer equal access and use of health care. Despite this welfare orientation, HCC has been reported to be one of Sweden's most socioeconomically polarized malignancies [22]. The incidence of HCC is seven times higher in the most economically deprived patients compared to the wealthiest [22]. Even after adjusting for other sociodemographic variables, etiology, liver disease severity, comorbidity, and BCLC stage, patients with a low household income had worse survival than the wealthiest patients. Here, we have shown that a low household income is also linked to a higher likelihood of having unrecognized cirrhosis, being diagnosed under non‐surveillance (despite recognition of cirrhosis), and having a late‐stage HCC at diagnosis.

Cirrhosis patients diagnosed with HCC under surveillance could have the most favorable prognosis, as they more often received treatments with curative intent. This assertion is consistent with data from a recent meta‐analysis and could lend support to implementing and developing surveillance strategies in patients with cirrhosis [5].

Although 55% of patients with recognized cirrhosis were diagnosed with HCC under surveillance, this proportion only accounted for 26% of all cases of HCC reported to the SweLiv between 2012 and 2018. This proportion of cases is in line with the proportion of surveilled patients in the United States [40] and what was reported in a recently published meta‐analysis [10]. The Region of Stockholm has reported similar results, where 22% of all cases were diagnosed by surveillance [41].

Undiagnosed liver disease and doctors’ failure to order surveillance were observed in a third of patients in Stockholm [41]. This finding is consistent with our sensitivity analysis showing that 28% of patients with known cirrhosis diagnosed without surveillance “should have been” diagnosed during surveillance but were not. The causes of this surveillance failure are unknown, as detailed surveillance data were not available in the SweLiv quality register. Most of these patients could have been excluded from surveillance programs due to cirrhosis decompensation, comorbidity, or lack of adherence. Some patients could have been excluded from surveillance by error, as reported in the Region of Stockholm [41]. Some might have been monitored but had aggressive tumors leading to HCC diagnosis between planned surveillance time points.

### Strength and limitations

We used a nationwide quality register (SweLiv) which comprised >95% of all adult individuals in Sweden diagnosed with HCC [18]. Thus, selection bias is minimized but cannot be ruled out. Additional nationwide registers used for this study are a source of high‐quality data and have been validated for cirrhosis and HCC [17, 23, 42]. Combining granular data from the SweLiv with other registers is a clear advantage as it captures more detailed data than ordinarily available in register‐based research. Despite a lack of data for some tumor‐ and patient‐related variables found in the SweLiv, we could classify patients into the two major groups of interest (early‐ and late‐stage HCC diagnosis) in 98% of the cases. We could also classify most patients as either compensated or decompensated. Likewise, surgical treatments reported in the SweLiv planned were confirmed in the NPR.

Free access to health care services in Sweden further reduces the risk of selection bias.

Nordic countries share several characteristics, e.g. free access to medical care of high level, free public education, and comprehensive welfare safety policies [43]. It is, however, unknown whether the associations we report also apply to other Nordic countries. Nevertheless, similarities between our results and observations from the United States indicate that a higher level of access to health care might not be sufficient to accurately identify patients with cirrhosis or sufficiently improve surveillance in patients with known cirrhosis.

This study has some limitations that provide suggestions for further research. Even with detailed data from the SweLiv, the main limitation concerns the lack of even more granular data. As noted earlier in this report, the NPR does not include data from primary care visits. Thus, some patients with unrecognized cirrhosis could have been misclassified. However, most patients diagnosed with cirrhosis in Sweden's primary care centers are often referred to secondary or tertiary hospitals.

The purpose of this study was not to estimate the effect of surveillance on survival. Thus, the potential impact of length and lead time bias must be considered when interpreting survival rate estimates [7, 44].

Length‐time bias infers that less aggressive tumors usually have a long asymptomatic period, making them more likely to be detected during surveillance [45]. Adjustments for length‐time bias can be achieved through statistical methods [45] but might also be avoided by including all surveilled patients into a single group, regardless of the diagnostic pathway [44]. Because surveillance is generally not recommended in patients with a high‐performance status or decompensation [3, 4], it would be plausible to assume that patients with known cirrhosis that are diagnosed without surveillance and signs of decompensation, as well as good performance status, should have been surveilled before HCC diagnosis. As described above, data from the SweLiv did not allow for identifying intention‐to‐surveil before HCC diagnosis in patients diagnosed with known cirrhosis without surveillance.

Lead time is the additional follow‐up time caused by the early detection of asymptomatic tumors under surveillance compared to patients with a similar tumor detected due to symptoms [45]. Different methods can achieve lead time adjustments, including those described by Duffy et al. [45]. As in the case of length time, our data did not allow for such adjustments. Some insights into the effect of lead time bias in estimated aHRs could be obtained by including the BCLC stage in the models.

In summary, adjustments for the BCLC stage could have reduced the effect of lead time bias in our models in that the HCC stage at diagnosis was intrinsically related to receipt of surveillance. We also performed sensitivity analyses to adjust for both length and lead time bias. We acknowledge, though, that our adjustments could be fractional approximations, and the possibility of residual length or lead time bias should be considered.

## Conclusions

Cirrhosis is often unrecognized in patients diagnosed with HCC. Unrecognized cirrhosis is associated with more advanced HCC at diagnosis and a worse prognosis. These findings underline the importance of the early detection of cirrhosis and inclusion in surveillance programs for timely HCC diagnosis.

Patients with unrecognized cirrhosis are more often male and are socioeconomically deprived. NAFLD and NAFLD‐related comorbidities, especially arterial hypertension and diabetes, are highly prevalent in patients with unrecognized cirrhosis. More efforts are needed to raise awareness of cirrhosis, not only for patients at high risk but also for clinical practitioners treating these patients.

## Conflict of interest

Nothing to report.

## Author contributions

Juan Andres Vaz: Conceptualization; Data curation; Formal analysis; Funding acquisition; Methodology; Visualization; Writing – original draft; Writing – review & editing. Ulf Strömberg: Conceptualization; Data curation; Formal analysis; Methodology; Supervision; Visualization; Writing – original draft; Writing – review & editing. Patrik Midlöv: Methodology; Supervision; Writing – original draft; Writing – review & editing. Berne Eriksson: Methodology; Supervision; Writing – review & editing. David Buchebner: Methodology; Supervision; Writing – review & editing. Hannes Hagström: Conceptualization; Formal analysis; Methodology; Supervision; Writing – original draft; Writing – review & editing.

## Supporting information


**Figure S1**. Swedish nationwide registers.
**Figure S2**. Classification of recognized and unrecognized cirrhosis in 2670 patients diagnosed with hepatocellular carcinoma (HCC) in Sweden between 2012 and 2018.
**Figure S3**. Swedish treatment algorithm for hepatocellular carcinoma.
**Figure S4**. Percentage of visits registered in the National Patient Register within 365 days before hepatocellular carcinoma (HCC) diagnosis for 735 patients with unrecognized cirrhosis, later diagnosed with HCC in Sweden between 2012 and 2018.
**Table S1**. List of variables.
**Table S2**. Baseline characteristics of patients with cirrhosis diagnosed with HCC in Sweden between 2012 and 2018.
**Table S3**. Baseline characteristics of patients with cirrhosis (surveilled vs. non‐surveilled) diagnosed with HCC in Sweden between 2012 and 2018.
**Table S4**. Factors associated with the likelihood of being diagnosed outside surveillance in patients with recognized and unrecognized cirrhosis compared to patients with recognized diagnosed with HCC under surveillance.
**Table S5**. Factors associated with the likelihood of having unrecognized liver cirrhosis before being diagnosed with HCC compared to patients with recognized cirrhosis diagnosed with HCC without surveillance.
**Table S6**. Different factors and their association with the likelihood of being diagnosed with HCC under non‐surveillance.
**Table S7**. Survival probabilities of patients with liver cirrhosis diagnosed with HCC in Sweden between 2012 and 2018.
**Table S8**. Univariable and multivariable estimates for overall mortality in 2139 patients with cirrhosis diagnosed with HCC in Sweden between 2012 and 2018.
