# Supplementary material for: Unrecognized liver cirrhosis is common and associated with worse survival in hepatocellular carcinoma: A nationwide cohort study of 3473 patients
Source: J Intern Med. 2022 Oct 3;293(2):184–99. doi: 10.1111/joim.13570 (PMC10091698; doi:10.1111/joim.13570)
Supplement: Supplementary file 1 — Figure S1. Swedish nationwide registers. Figure S2. Classification of recognized and unrecognized cirrhosis in 2670 patients diagnosed with hepatocellular carcinoma (HCC) in Sweden between 2012 and 2018. Figure S3. Swedish treatment algorithm for hepatocellular carcinoma. Figure S4. Percentage of visits registered in the National Patient Register within 365 days before hepatocellular carcinoma (HCC) diagnosis for 735 patients with unrecognized cirrhosis, later diagnosed with HCC in Sweden between 2012 and 2018. Table S1. List of variables. Table S2. Baseline characteristics of patients with cirrhosis diagnosed with HCC in Sweden between 2012 and 2018. Table S3. Baseline characteristics of patients with cirrhosis (surveilled vs. non‐surveilled) diagnosed with HCC in Sweden between 2012 and 2018. Table S4. Factors associated with the likelihood of being diagnosed outside surveillance in patients with recognized and unrecognized cirrhosis compared to patients with recognized diagnosed with HCC under surveillance. Table S5. Factors associated with the likelihood of having unrecognized liver cirrhosis before being diagnosed with HCC compared to patients with recognized cirrhosis diagnosed with HCC without surveillance. Table S6. Different factors and their association with the likelihood of being diagnosed with HCC under non‐surveillance. Table S7. Survival probabilities of patients with liver cirrhosis diagnosed with HCC in Sweden between 2012 and 2018. Table S8. Univariable and multivariable estimates for overall mortality in 2139 patients with cirrhosis diagnosed with HCC in Sweden between 2012 and 2018. [file JOIM-293-184-s001.docx]

**Unrecognized liver cirrhosis is common and associated with worse survival in hepatocellular carcinoma: A nationwide cohort study of 3,473 patients**

Juan Vaz, Ulf Strömberg, Patrik Midlöv, Berne Eriksson, David Buchebner, Hannes Hagström

**SUPPLEMENTARY MATERIAL**

**Table of contents**

Supporting tables

Table S1. List of variables ……………………………………………………………………………………………………………………………………………………………………………………………………………. 3

Table S2. Baseline characteristics of patients with cirrhosis diagnosed with HCC in Sweden between 2012 and 2018 ……………………………………………………………………. 7

Table S3. Baseline characteristics of patients with cirrhosis (surveilled vs. non-surveilled) diagnosed with HCC in Sweden between 2012 and 2018 ……………………. 10

Table S4. Factors associated with the likelihood of being diagnosed outside surveillance in patients with recognized and unrecognized cirrhosis compared to

patients with recognized diagnosed with HCC under surveillance ………………………………………………………………………………………………………………………………………………. 13

Table S5. Factors associated with the likelihood of having unrecognized liver cirrhosis before being diagnosed with HCC compared to patients with recognized

cirrhosis diagnosed with HCC without surveillance ……………………………………………………………………………………………………………………………………………………………………. 14

Table S6. Different factors and their association with the likelihood of being diagnosed with HCC under non-surveillance …………………………………………………………… 15

Table S7. Survival probabilities of patients with liver cirrhosis diagnosed with HCC in Sweden between 2012 and 2018 ……………………………………………………………… 16

Table S8. Univariable and multivariable estimates for overall mortality in 2139 patients with cirrhosis diagnosed with HCC in Sweden between 2012 and 2018 …. 18

SUPPORTING FIGURES

Figure S1. Swedish nationwide registers ……………………………………………………………………………………………………………………………………………………………………………………. 19

Figure S2. Classification of recognized and unrecognized cirrhosis in 2670 patients diagnosed with hepatocellular carcinoma (HCC) in Sweden between 2012 and

2018 ………………………………………………………………………………………………………………………………………………………………………………………………………………………………………… 20

Figure S3. Swedish treatment algorithm for hepatocellular carcinoma ………………………………………………………………………………………………………………………………………... 21

Figure S4. Percentage of visits registered in the National Patient Register within 365 days before hepatocellular carcinoma (HCC) diagnosis for 735 patients with

unrecognized cirrhosis, later diagnosed with HCC in Sweden between 2012 and 2018 ………………………………………………………………………………………………………………... 22

| **Table S1. List of variables** | | | | | | | | | | | |
| --- | --- | --- | --- | --- | --- | --- | --- | --- | --- | --- | --- |
| **Variable** | **Definition** | | | | | | | | | | **Source** |
| Sociodemographic  characteristics | | | | | | | | | | | |
| Sex | Sex according to the Swedish population register | | | | | | | | | | Statistics Sweden |
|  |  | | | | | | | | | |  |
| Age at diagnosis | Age in years | | | | | | | | | | SweLiv |
|  |  | | | | | | | | | |  |
| Country of birth | Nordic: Sweden, Norway, Finland, Denmark, or Iceland | | | | | | | | | | Statistics Sweden |
|  | Non-Nordic: country not listed above | | | | | | | | | |  |
|  |  | | | | | | | | | |  |
| Household income | Low: <25% of mean household income for all households in Sweden | | | | | | | | | | Statistics Sweden |
|  | Medium: 25-75% of mean household income for all households in Sweden | | | | | | | | | |  |
|  | High: >75% of mean household income for all households in Sweden | | | | | | | | | |  |
| Liver disease, aetiology | | | | | | | | | | | |
| Alcohol | ICD-10-SE: E52, F10-F10.9, K29.2, K70.0, K70.1, K70.2, K70.3, K70.4, K70.9, K85.2, K86.0, R78.0, Y90 | | | | | | | | | | NPR |
|  | *AND/OR* | | | | | | | | | |  |
|  | ATC: N07BB01 (disulfiram), N07BB03 (acamprosat), N07BB04 (naltrexon) | | | | | | | | | | PDR |
|  |  | | | | | | | | | |  |
| Viral hepatitis | ICD-10-SE: B18.2 (A-X), B18.0 (F-H), B18.1 (A-X), B18.8 (A-X), B18.9 (A-X), B19 (0-9) | | | | | | | | | | NPR |
|  |  | | | | | | | | | |  |
| Primary sclerosing cholangitis | ICD-10-SE: K83.0A | | | | | | | | | | NPR |
|  |  | | | | | | | | | |  |
| Primary biliary cholangitis | ICD-10-SE: K74.3 | | | | | | | | | | NPR |
|  | *AND/OR* | | | | | | | | | |  |
|  | ATC: A05AA02 (ursodeoxycholic acid) | | | | | | | | | | PDR |
|  |  | | | | | | | | | |  |
| Autoimmune hepatitis | ICD-10-SE: K75.4 | | | | | | | | | | NPR |
|  |  | | | | | | | | | |  |
| Other | ICD-10-SE: E80.0, E83.0B, E83.1, E88.0 (A-B), K76.5 | | | | | | | | | | NPR |
|  |  | | | | | | | | | |  |
| Cryptogenic | ICD-10-SE: K74.6 | | | | | | | | | | NPR |
|  | *AND* | | | | | | | | | |  |
|  | No other cause of cirrhosis | | | | | | | | | | NPR/SweLiv |
|  |  | | | | | | | | | |  |
| Non-alcoholic fatty liver | ICD-10-SE: K75.8, K76.0 | | | | | | | | | | NPR |
| disease | *AND/OR* | | | | | | | | | |  |
|  | Obesity (see below) | | | | | | | | | | NPR |
|  | *AND/OR* | | | | | | | | | |  |
|  | Diabetes mellitus (see below) | | | | | | | | | | NPR/PDR |
|  | *AND* | | | | | | | | | |  |
|  | No other cause of cirrhosis | | | | | | | | | | NPR/SweLiv |
| Liver cirrhosis | | | | | | | | | | | |
|  | ICD-10-SE: B18.0E, B18.0G, B18.1E, B18.1G, B18.2E, B18.2G, B18.8E, B18.8G, B18.9E, B18.9G, G93.4, I85.0 I85.9, I98.2, I98.3, K70.3, | | | | | | | | | | NPR |
|  | K71.7, K74.6, K76.6, K76.7 | | | | | | | | | |  |
|  | *AND/OR* | | | | | | | | | |  |
|  | Registered by reporting physician | | | | | | | | | | SweLiv |
| Comorbidity | | | | | | | | | | | |
| Obesity | ICD-10-SE: E66.0-9 | | | | | | | | | | NPR |
|  |  | | | | | | | | | |  |
| Diabetes mellitus | ICD-10-SE: E10-E14 | | | | | | | | | | NPR |
|  | *AND/OR* | | | | | | | | | |  |
|  | ATC: A10AB-A10AE (insulin), A10BA (biguanides), A10BB-A10BX (other glucose-lowering drugs) | | | | | | | | | | PDR |
|  |  | | | | | | | | | |  |
| Arterial hypertension | ICD-10-SE: I10-I15 | | | | | | | | | | NPR |
|  | *AND/OR* | | | | | | | | | |  |
|  | ATC: C02A-C02N, C03AA (hydrochloride), C03EA (+ kalium), C07AB (selective beta-blockers), C07FB02 (metoprolol and felodipin), C08CA01 (amlodipin), C08CA02 (felodipin), C09A-C09B (ACE-inhibitors and combinations), C09C-C09D (ARB and combinations) | | | | | | | | | | PDR |
|  |  | | | | | | | | | |  |
| Coronary artery disease | ICD-10-SE: I20-I25 | | | | | | | | | | NPR |
|  | *AND/OR* | | | | | | | | | |  |
|  | ATC: C01DA (organic nitrates) | | | | | | | | | | PDR |
| Tumor  characteristics | | | | | | | | | | | |
| Tumor size | Largest tumour (mm) | | | | | | | | | | SweLiv |
|  | Missing: 244 (9%) | | | | | | | | | |  |
|  |  | | | | | | | | | |  |
| Number of tumors | Single, 2-3, or >3 (multinodular) | | | | | | | | | | SweLiv |
|  | Missing: 381 (14%) | | | | | | | | | |  |
|  |  | | | | | | | | | |  |
| Portal vein thrombosis | Yes/No. No missing data | | | | | | | | | | SweLiv |
| Alpha-fetoprotein | Measured in μg/L | | | | | | | | | | SweLiv |
|  | Missing: 834 (31%) | | | | | | | | | |  |
|  |  | | | | | | | | | |  |
| Regional metastasis | TNM classification for HCC 8^th^ Edition: N0, N1, NX | | | | | | | | | | SweLiv |
|  | Missing: 31 (1%) | | | | | | | | | |  |
|  |  | | | | | | | | | |  |
| Distant metastasis | TNM classification for HCC 8^th^ Edition: M0, M1, MX | | | | | | | | | | SweLiv |
|  | Missing: 109 (4%) | | | | | | | | | |  |
| ECOG PS | | | | | | | | | | | |
|  | 0: Fully active, able to carry on all pre-disease performance without restriction. | | | | | | | | | | SweLiv |
|  | 1: Restricted in physically strenuous activity but ambulatory and able to perform work of a light or sedentary nature. | | | | | | | | | |  |
|  | 2: Ambulatory and capable of all self-care but unable to carry out any work activities; up and about >50% of waking hours. | | | | | | | | | |  |
|  | 3: Capable of only limited self-care; confined to bed or chair >50% of waking hours. | | | | | | | | | |  |
|  | 4: Completely disabled; cannot conduct any self-care; totally confined to bed or chair. | | | | | | | | | |  |
|  |  | | | | | | | | | |  |
|  | Missing: 513 (15%) | | | | | | | | | |  |
| Child-Pugh score | | | | | | | | | | | |
|  |  | |  | |  | |  | |  | |  |
|  | Bilirubin (μmol/L) | | Albumin (g/L) | | INR | | Ascites | | Encephalopathy | | SweLiv |
|  | < 34.2 | +1 | > 35 | +1 | < 1.7 | +1 | Absent | +1 | None | +1 |  |
|  | 34.2-51.3 | +2 | 28-35 | +2 | 1.7-2.2 | +2 | Slight | +2 | Gr. 1-2 | +2 |  |
|  | > 51.3 | +3 | < 28 | +3 | > 2.2 | +3 | Moderate | +3 | Gr. 3-4 | +3 |  |
|  |  | | | | | | | | | |  |
|  | Missing data: | | | | | | | | | |  |
|  | Bilirubin: 646 (19%) | | | | | | | | | |  |
|  | Albumin: 726 (21%) | | | | | | | | | |  |
|  | INR: 717 (21%) | | | | | | | | | |  |
|  | Ascites: 385 (11%) | | | | | | | | | |  |
|  | Encephalopathy: 520 (15%) | | | | | | | | | |  |
|  |  | | | | | | | | | |  |
|  | Child-Pugh score calculation for missing data: | | | | | | | | | |  |
|  | 1 laboratory variable: CP ≤ 7 if the sum of known variables = 4 | | | | | | | | | |  |
|  | 2-3 laboratory variables: CP ≥ 7 if the sum of known variables ≥ 6 | | | | | | | | | |  |
|  | Encephalopathy grade: +1 if the sum of known variables = 4 and ECOG = 0 | | | | | | | | | |  |
|  | Ascites grade: +1 if the sum of known variables = 4 and ECOG = 0 | | | | | | | | | |  |
| Diagnostic pathway | | | | | | | | | | | |
|  | Surveillance: patient with known liver disease included in the surveillance program for HCC | | | | | | | | | | SweLiv, NPR |
|  | Clinical symptoms: diagnosed with HCC during a clinical work-up related to liver disease-associated symptoms | | | | | | | | | |  |
|  | Incidental: HCC diagnosis on passant in surgery or radiology in a patient without clinical symptoms related to liver disease | | | | | | | | | |  |
|  |  | | | | | | | | | |  |
|  | Diagnostic pathway for missing data: | | | | | | | | | |  |
|  | Clinical symptoms: if no prior liver disease was registered and ICD-10-SE codes such as R18 (ascites) or R10.0 (abdominal pain), | | | | | | | | | |  |
|  | among others, were registered at the time of HCC diagnosis | | | | | | | | | |  |
|  | Incidental: if no prior liver disease was registered and no other symptom-related ICD-10-SE codes were registered at the time of HCC | | | | | | | | | |  |
|  | diagnosis | | | | | | | | | |  |
| Barcelona Clinic Liver  Cancer stage | | | | | | | | | | | |
|  | Early (0-A) – criteria: | | | | | | | | | | SweLiv |
|  | 1) Single tumor or up to 3 nodules ≤3 cm *AND* | | | | | | | | | |  |
|  | 2) N0, M0 *AND* | | | | | | | | | |  |
|  | 3) ECOG PS 0-1 (2 in transplant/resection candidates) *AND* | | | | | | | | | |  |
|  | 4) Child-Pugh score ≤7 (not necessary if transplant candidate) | | | | | | | | | |  |
|  |  | | | | | | | | | |  |
|  | Late (B-D) – criteria (at least one) | | | | | | | | | |  |
|  | 1) 2-3 nodules of with one with size >3 cm *OR* | | | | | | | | | |  |
|  | 2) Multinodular *OR* | | | | | | | | | |  |
|  | 3) ECOG PS >2 *OR* | | | | | | | | | |  |
|  | 4) N1 *OR* | | | | | | | | | |  |
|  | 5) M1 *OR* | | | | | | | | | |  |
|  | 6) Portal vein thrombosis/tumor thrombosis *OR* | | | | | | | | | |  |
|  | 7) Child-Pugh score > 7 (if not transplant candidate and conditions for early HCC not fulfilled) | | | | | | | | | |  |
|  |  | | | | | | | | | |  |
|  | HCC-stage classification for missing data: | | | | | | | | | |  |
|  | Number of tumors: late-stage if late-stage criteria 2-7 fulfilled; unknown if criteria for early-stage otherwise fulfilled | | | | | | | | | |  |
|  | Tumor size: non-relevant for single tumors; if late-stage criteria 2-7 are fulfilled and the patient is not a transplant candidate | | | | | | | | | |  |
|  | N or M: early-stage if criteria for early-stage otherwise fulfilled and patient candidate for resection/transplant; late-stage | | | | | | | | | |  |
|  | of criteria for late-stage otherwise fulfilled | | | | | | | | | |  |
|  | ECOG PS: early-stage if criteria for early-stage are otherwise fulfilled and patient candidate for resection/transplant; late-stage if criteria for late-stage are otherwise fulfilled | | | | | | | | | |  |
| Treatment | | | | | | | | | | | |
|  | Transplantation: if registered at any point, regardless of previous treatments | | | | | | | | | | SweLiv/NPR |
|  | Resection: if performed before ablation | | | | | | | | | |  |
|  | Ablation: if performed before resection | | | | | | | | | |  |
|  |  | | | | | | | | | |  |
|  | Transarterial chemoembolization | | | | | | | | | | SweLiv |
|  | Systemic chemotherapy: ATC L01EX02 (sorafenib) *AND/OR* ATC L01EX05 (regorafenib) | | | | | | | | | | PDR |
|  |  | | | | | | | | | |  |
|  | Best supportive care: no treatment reported | | | | | | | | | | SweLiv/NPR/PDR |
| Number of visits | | | | | | | | | | | |
|  | Number of entries found in NPR within 365 days before HCC diagnosis | | | | | | | | | | NPR |
| ATC: Anatomical Therapeutic Chemical Classification System; ECOG PS = Eastern Cooperative Oncology Group performance status; HCC = Hepatocellular carcinoma; Household income = Disposable income per household per consumption unit; ICD-10-SE: International Classification of Diseases 10^th^ Revision - Swedish Edition; NPR: National Patient Register; PDR: Prescribed Drug Register; INR = International Normalized Ratio; M: Distant metastasis; N: Regional lymph node metastasis. SweLiv: Swedish quality register for cancers found in the liver, gallbladder and bile ducts.  Variables registered at the time of HCC diagnosis if not otherwise stated. No missing values if otherwise stated.  Household income refers to a person’s disposable income per consumption unit. Disposable income is the sum of all taxable and tax-free income minus taxes and negative transfers. The income includes gains/losses, i.e. the gain/loss arising from a sale (realization) of assets, for example, stocks, mutual funds or real estate. To compare disposable income and economical purchasing power between different household types, a weight system is used where consumption is related to household composition. Disposable income is divided by the weight of consumption of the household. The scale is determined by Statistics Sweden and is based on, among other things, budget calculations carried out by the Swedish Consumer Agency and the basis for assessing a basic consumption that can be calculated for different household types. The consumption unit scale used by Statistics Sweden is the following: single or living alone 1.0; cohabiting couple 1.51; additional adult 0.6; first child 0-19 years 0.52; second and subsequent children 0-19 years 0.42. | | | | | | | | | | | |

| **Table S2.** Baseline characteristics of patients with cirrhosis diagnosed with HCC in Sweden between 2012 and 2018 | | | | | | | | |
| --- | --- | --- | --- | --- | --- | --- | --- | --- |
|  | **Overall** | **Recognized LC** | |  |  | **P-value** | | |
|  |  | **Surveillance (A)** | **No surveillance (B)** | **Unrecognized LC (C)** |  | **A vs. B** | **A vs. C** | **B vs. C** |
| **Total, n (%)** | 2670 (100) | 901 (34) | 736 (27) | 1033 (39) |  | - | - | - |
| **Sex (male)** | 2082 (78) | 684 (76) | 543 (74) | 855 (83) |  | 0.330 | <0.001 | <0.001 |
| **Median age (years)** | 67 (61-74) | 65 (59-71) | 68 (61-74) | 69 (62-76) |  | <0.001 | <0.001 | <0.001 |
| **Country of birth** |  |  |  |  |  |  |  |  |
| Nordic | 2243 (84) | 740 (82) | 636 (86) | 867 (84) |  | 0.021 | 0.302 | 0.157 |
| Non-Nordic | 427 (16) | 161 (18) | 100 (14) | 166 (16) |  |  |  |  |
| **Household income** |  |  |  |  |  |  |  |  |
| High | 361 (14) | 140 (16) | 99 (14) | 122 (12) |  | 0.260 | 0.005 | 0.630 |
| Medium | 1053 (39) | 379 (42) | 268 (36) | 406 (39) |  | 0.022 | 0.228 | 0.233 |
| Low | 1256 (47) | 383 (42) | 369 (50) | 505 (49) |  | 0.002 | 0.020 | 0.308 |
| **Etiology** |  |  |  |  |  |  |  |  |
| Viral hepatitis | 550 (21) | 233 (26) | 117 (16) | 200 (19) |  | <0.001 | <0.001 | 0.068 |
| Viral hepatitis + ALD | 586 (22) | 258 (29) | 167 (22) | 161 (16) |  | 0.007 | <0.001 | <0.001 |
| ALD | 595 (22) | 176 (19) | 196 (27) | 223 (22) |  | <0.001 | 0.285 | 0.015 |
| NAFLD | 420 (16) | 87 (10) | 108 (15) | 225 (22) |  | 0.002 | <0.001 | <0.001 |
| Other | 279 (10) | 105 (12) | 111 (15) | 63 (6) |  | 0.041 | <0.001 | <0.001 |
| Cryptogenic | 240 (9) | 42 (4) | 37 (5) | 161 (16) |  | 0.730 | <0.001 | <0.001 |
| **Decompensation**^a^ | 1016 (38) | 225 (25) | 417 (57) | 374 (36) |  | <0.001 | <0.001 | <0.001 |
| **Comorbidity** |  |  |  |  |  |  |  |  |
| Arterial hypertension | 1490 (856) | 484 (54) | 421 (58) | 585 (57) |  | 0.162 | 0.200 | 0.846 |
| Type 2 diabetes | 1090 (41) | 338 (38) | 331 (45) | 421 (41) |  | 0.002 | 0.148 | 0.079 |
| CAD | 407 (15) | 93 (10) | 130 (18) | 184 (18) |  | <0.001 | <0.001 | 0.950 |
| **N of visits to the NPR**^b^ | 3 (1-6) | 3 (2-7) | 4 (2-7) | 2 (1-4) |  | 0.489 | <0.001 | <0.001 |
| **Diagnostic pathway** |  |  |  |  |  |  |  |  |
| Surveillance | 901 (34) | 901 (100) | 0 | 0 |  | - | - | - |
| Clinical | 1397 (52) | 0 | 582 (79) | 815 (79) |  | - | - | 0.953 |
| Incidental | 372 (14) | 0 | 154 (21) | 218 (21) |  | - | - | 0.086 |
| **Ascites** |  |  |  |  |  |  |  |  |
| Absent | 1739 (65) | 690 (77) | 378 (51) | 671 (65) |  | <0.001 | <0.001 | <0.001 |
| Slight | 332 (12) | 102 (11) | 115 (16) | 115 (11) |  | 0.013 | 0.942 | 0.006 |
| Moderate | 321 (12) | 50 (5) | 163 (22) | 108 (11) |  | <0.001 | <0.001 | <0.001 |
| Unknown | 278 (11) | 59 (7) | 80 (11) | 139 (13) |  | 0.002 | <0.001 | 0.108 |
| **Encephalopathy** |  |  |  |  |  |  |  |  |
| Absent | 2174 (81) | 811 (90) | 545 (74) | 818 (79) |  | <0.001 | <0.001 | 0.012 |
| Grade 1-2 | 79 (3) | 20 (2) | 45 (6) | 14 (1) |  | <0.001 | 0.167 | <0.001 |
| Grade 3-4 | 24 (1) | 3 (<<1) | 14 (2) | 7 (<1) |  | 0.002 | 0.354 | 0.025 |
| Unknown | 393 (15) | 69 (8) | 132 (18) | 194 (19) |  | <0.001 | <0.001 | 0.664 |
| **Albumin (g/L)** |  |  |  |  |  |  |  |  |
| Median | 32 (27-36) | 34 (30-38) | 29 (25-33) | 31 (26-36) |  | <0.001 | <0.001 | <0.001 |
| Unknown | 512 (19) | 152 (17) | 135 (18) | 225 (22) |  | 0.472 | 0.007 | 0.082 |
| **Bilirubin (μmol/L)** |  |  |  |  |  |  |  |  |
| Median | 17 (11-31) | 16 (10-26) | 22 (13-42) | 15 (10-27) |  | <0.001 | 0.086 | <0.001 |
| Unknown | 460 (17) | 147 (16) | 120 (16) | 193 (19) |  | 1.000 | 0.188 | 0.206 |
| **PT (INR)** |  |  |  |  |  |  |  |  |
| Median | 1.2 (1.1-1.4) | 1.2 (1.1-1.3) | 1.3 (1.1-1.4) | 1.2 (1.1-1.3) |  | <0.001 | 0.482 | <0.001 |
| Unknown | 504 (19) | 151 (17) | 134 (18) | 219 (21) |  | 0.471 | 0.015 | 0.131 |
| **Performance status** |  |  |  |  |  |  |  |  |
| **(ECOG)** |  |  |  |  |  |  |  |  |
| 0 | 936 (35) | 484 (54) | 191 (26) | 261 (25) |  | <0.001 | <0.001 | 0.782 |
| 1 | 634 (24) | 239 (27) | 159 (22) | 236 (23) |  | 0.024 | 0.064 | 0.563 |
| ≥2 | 708 (27) | 101 (11) | 259 (35) | 348 (34) |  | <0.001 | <0.001 | 0.542 |
| Unknown | 392 (15) | 77 (8) | 127 (17) | 188 (18) |  | <0.001 | <0.001 | 0.615 |
| **AFP (μg/L)** |  |  |  |  |  |  |  |  |
| Median | 17 (5-405) | 10 (5-64) | 22 (6-853) | 37 (6-1128) |  | <0.001 | <0.001 | 0.206 |
| Unknown | 824 (31) | 260 (29) | 243 (33) | 321 (31) |  | 0.076 | 0.297 | 0.408 |
| **Tumor size (mm)** |  |  |  |  |  |  |  |  |
| Median | 40 (25-70) | 29 (40-65) | 55 (34-95) | 55 (34-95) |  | <0.001 | <0.001 | <0.001 |
| <20 | 494 (18) | 266 (30) | 119 (16) | 109 (11) |  | <0.001 | <0.001 | <0.001 |
| 20-29 | 471 (18) | 247 (27) | 122 (17) | 102 (10) |  | <0.001 | <0.001 | <0.001 |
| ≥30 | 1461 (55) | 354 (39) | 400 (54) | 707 (68) |  | <0.001 | <0.001 | <0.001 |
| Unknown | 244 (9) | 34 (4) | 95 (13) | 115 (11) |  | <0.001 | <0.001 | 0.264 |
| **Number of tumors** |  |  |  |  |  |  |  |  |
| 1 | 1211 (46) | 483 (54) | 310 (42) | 418 (41) |  | <0.001 | <0.001 | 0.493 |
| 2-3 | 705 (26) | 279 (31) | 197 (27) | 229 (22) |  | 0.063 | <0.001 | 0.028 |
| >3 | 373 (14) | 92 (10) | 96 (13) | 185 (18) |  | 0.086 | <0.001 | 0.006 |
| Unknown | 381 (14) | 47 (5) | 133 (18) | 201 (19) |  | <0.001 | <0.001 | 0.498 |
| **Metastasis** |  |  |  |  |  |  |  |  |
| Regional | 307 (12) | 49 (6) | 78 (11) | 180 (18) |  | <0.001 | <0.001 | <0.001 |
| Extrahepatic | 374 (15) | 37 (4) | 112 (16) | 225 (23) |  | <0.001 | <0.001 | <0.001 |
| **PVT** | 549 (21) | 82 (9) | 174 (24) | 293 (29) |  | <0.001 | <0.001 | 0.042 |
| **BCLC-stage** |  |  |  |  |  |  |  |  |
| 0-A | 795 (30) | 456 (51) | 144 (20) | 195 (19) |  | <0.001 | <0.001 | 0.714 |
| B-D | 1818 (68) | 414 (46) | 583 (79) | 821 (79) |  | <0.001 | <0.001 | 0.905 |
| Missing | 57 (2) | 31 (3) | 9 (1) | 17 (2) |  | 0.004 | 0.013 | 0.550 |
| **Treatment** |  |  |  |  |  |  |  |  |
| Transplantation | 225 (9) | 137 (15) | 54 (7) | 34 (3) |  | <0.001 | <0.001 | <0.001 |
| Resection | 322 (12) | 158 (18) | 47 (6) | 117 (11) |  | <0.001 | <0.001 | <0.001 |
| Ablation | 456 (17) | 276 (31) | 87 (12) | 93 (9) |  | <0.001 | <0.001 | 0.056 |
| Palliative | 572 (21) | 169 (18) | 145 (20) | 258 (25) |  | <0.001 | <0.001 | 0.459 |
| BSC | 1095 (41) | 161 (18) | 403 (55) | 531 (52) |  | <0.001 | <0.001 | 0.176 |
| AFP: Alpha-fetoprotein; ALD: Alcoholic liver disease; BCLC: Barcelona Clinic Liver Cancer staging system; BSC: Best supportive care; CAD: Coronary artery disease; ECOG: Eastern Cooperative Oncology Group; HCC: Hepatocellular carcinoma; NAFLD: Non-alcoholic fatty liver disease; NPR: National Patient Registry; PT (INR): Prothrombin time (international normalized ratio); PVT: Portal vein thrombosis. Nordic country of birth: Sweden, Denmark, Finland, Iceland, and Norway. Household income is defined as disposable income per household per consumption unit. Median age, tumor size, and laboratory values presented with corresponding interquartile range in parenthesis. A p-value <0.05 from a two-tailed Chi-square (categorical variables), or Mann-Whitney U test (continuous variables) was considered statistically significant. Statistically significant results are highlighted in grey (non-clinically relevant associations were not highlighted). ^a^ Decompensated cirrhosis is defined as ascites or encephalopathy or bilirubin≥52 μmol/L or albumin<28 g/L. ^b^ Registered within 365 days before HCC diagnosis. | | | | | | | | |

| **Table S3.** Baseline characteristics of patients with cirrhosis (surveilled vs. non-surveilled) diagnosed with HCC in Sweden between 2012 and 2018 | | | | |
| --- | --- | --- | --- | --- |
|  | **Overall** | **Surveillance** | **No surveillance** | **P-value** |
| **Total, n (%)** | 2670 (100) | 901 (34) | 1769 (66) |  |
| **Sex (male)** | 2082 (78) | 684 (76) | 1398 (79) | 0.068 |
| **Median age (years)** | 67 (61-74) | 65 (59-71) | 69 (61-75) | <0.001 |
| **Country of birth** |  |  |  |  |
| Nordic | 2243 (84) | 740 (82) | 1503 (85) | 0.065 |
| Non-Nordic | 427 (16) | 161 (18) | 266 (15) |  |
| **Household income** |  |  |  |  |
| High | 361 (14) | 140 (16) | 221 (13) | 0.031 |
| Medium | 1053 (39) | 379 (42) | 674 (38) | 0.049 |
| Low | 1256 (47) | 383 (42) | 874 (49) | <0.001 |
| **Etiology** |  |  |  |  |
| Viral hepatitis | 550 (21) | 233 (26) | 317 (18) | <0.001 |
| Viral hepatitis + ALD | 586 (22) | 258 (29) | 328 (18) | <0.001 |
| ALD | 595 (22) | 176 (19) | 419 (24) | 0.016 |
| NAFLD | 420 (16) | 87 (10) | 333 (19) | <0.001 |
| Other | 279 (10) | 105 (12) | 174 (10) | 0.160 |
| Cryptogenic | 240 (9) | 42 (4) | 198 (11) | <0.001 |
| **Decompensation**^a^ | 1016 (38) | 225 (25) | 791 (45) | <0.001 |
| **Comorbidity** |  |  |  |  |
| Arterial hypertension | 1490 (856) | 484 (54) | 1006 (57) | 0.127 |
| Type 2 diabetes | 1090 (41) | 338 (38) | 752 (43) | 0.014 |
| CAD | 407 (15) | 93 (10) | 314 (18) | <0.001 |
| **N of visits to the NPR**^b^ | 3 (1-6) | 3 (2-7) | 2 (1-5) | <0.001 |
| **Ascites** |  |  |  |  |
| Absent | 1739 (65) | 690 (77) | 1049 (59) | <0.001 |
| Slight | 332 (12) | 102 (11) | 230 (13) | 0.239 |
| Moderate | 321 (12) | 50 (5) | 271 (16) | <0.001 |
| Unknown | 278 (11) | 59 (7) | 219 (12) | <0.001 |
| **Encephalopathy** |  |  |  |  |
| Absent | 2174 (81) | 811 (90) | 1363 (77) | <0.001 |
| Grade 1-2 | 79 (3) | 20 (2) | 59 (3) | 0.117 |
| Grade 3-4 | 24 (1) | 3 (<<1) | 21 (1) | 0.029 |
| Unknown | 393 (15) | 69 (8) | 132 (18) | <0.001 |
| **Albumin (g/L)** |  |  |  |  |
| Median | 32 (27-36) | 34 (30-38) | 30 (26-35) | <0.001 |
| Unknown | 512 (19) | 152 (17) | 360 (20) | 0.033 |
| **Bilirubin (μmol/L)** |  |  |  |  |
| Median | 17 (11-31) | 16 (10-26) | 18 (11-33) | 0.063 |
| Unknown | 460 (17) | 147 (16) | 313 (18) | 0.386 |
| **PT (INR)** |  |  |  |  |
| Median | 1.2 (1.1-1.4) | 1.2 (1.1-1.3) | 1.2 (1.1-1.4) | 0.003 |
| Unknown | 504 (19) | 151 (17) | 353 (20) | 0.047 |
| **Performance status** |  |  |  |  |
| **(ECOG)** |  |  |  |  |
| 0 | 936 (35) | 484 (54) | 452 (26) | <0.001 |
| 1 | 634 (24) | 239 (27) | 395 (22) | 0.018 |
| ≥2 | 708 (27) | 101 (11) | 607 (34) | <0.001 |
| Unknown | 392 (15) | 77 (8) | 315 (18) | <0.001 |
| **AFP (μg/L)** |  |  |  |  |
| Median | 17 (5-405) | 10 (5-64) | 29 (6-1000) | <0.001 |
| Unknown | 824 (31) | 260 (29) | 564 (32) | 0.111 |
| **Tumor size (mm)** |  |  |  |  |
| Median | 40 (25-70) | 29 (40-65) | 50 (30-80) | <0.001 |
| <20 | 494 (18) | 266 (30) | 228 (13) | <0.001 |
| 20-29 | 471 (18) | 247 (27) | 224 (13) | <0.001 |
| ≥30 | 1461 (55) | 354 (39) | 1107 (62) | <0.001 |
| Unknown | 244 (9) | 34 (4) | 210 (12) | <0.001 |
| **Number of tumors** |  |  |  |  |
| 1 | 1211 (46) | 483 (54) | 728 (41) | <0.001 |
| 2-3 | 705 (26) | 279 (31) | 426 (24) | <0.001 |
| >3 | 373 (14) | 92 (10) | 281 (16) | <0.001 |
| Unknown | 381 (14) | 47 (5) | 334 (19) | <0.001 |
| **Metastasis** |  |  |  |  |
| Regional | 307 (12) | 49 (6) | 258 (15) | <0.001 |
| Extrahepatic | 374 (15) | 37 (4) | 337 (19) | <0.001 |
| **PVT** | 549 (21) | 82 (9) | 467 (26) | <0.001 |
| **BCLC-stage** |  |  |  |  |
| 0-A | 795 (30) | 456 (51) | 339 (19) | <0.001 |
| B-D | 1818 (68) | 414 (46) | 1404 (79) | <0.001 |
| Missing | 57 (2) | 31 (3) | 26 (2) | 0.002 |
| **Treatment** |  |  |  |  |
| Transplantation | 225 (9) | 137 (15) | 88 (5) | <0.001 |
| Resection | 322 (12) | 158 (18) | 164 (9) | <0.001 |
| Ablation | 456 (17) | 276 (31) | 180 (10) | <0.001 |
| Palliative | 572 (21) | 169 (18) | 403 (23) | <0.001 |
| BSC | 1095 (41) | 161 (18) | 934 (53) | <0.001 |
| AFP: Alpha-fetoprotein; ALD: Alcoholic liver disease; BCLC: Barcelona Clinic Liver Cancer staging system; BSC: Best supportive care; CAD: Coronary artery disease; ECOG: Eastern Cooperative Oncology Group; HCC: Hepatocellular carcinoma; NAFLD: Non-alcoholic fatty liver disease; NPR: National Patient Registry; PT (INR): Prothrombin time (international normalized ratio); PVT: Portal vein thrombosis. No surveillance comprised patients with known cirrhosis diagnosed without surveillance and patients with priorly unrecognized cirrhosis. Nordic country of birth: Sweden, Denmark, Finland, Iceland and Norway. Household income is defined as disposable income per household per consumption unit. Median age, tumor size, and laboratory values presented with corresponding interquartile range in parenthesis. A p-value <0.05 from a two-tailed chi-square test (categorical variables), or Mann-Whitney U test (continuous variables) was considered statistically significant. Statistically significant results are highlighted in grey (non-clinically relevant associations were not highlighted). ^a^ Decompensated cirrhosis is defined as ascites or encephalopathy or bilirubin ≥52 μmol/L or albumin<28 g/L. ^b^ Registered within 365 days before HCC diagnosis. | | | | |

| **Table S4.** Factors associated with the likelihood of being diagnosed outside surveillance in patients with recognized and unrecognized cirrhosis compared to patients with recognized diagnosed with HCC under surveillance | | | | | | | | | |
| --- | --- | --- | --- | --- | --- | --- | --- | --- | --- |
|  | **Recognized cirrhosis (no surveillance)** | | | |  | **Unrecognized cirrhosis** | | | |
|  | **Univariable 1** | | **Multivariable 1** | |  | **Univariable 2** | | **Multivariable 2** | |
|  | OR (95% CI) | P-value | aOR (95% CI) | P-value |  | OR (95% CI) | P-value | aOR (95% CI) | P-value |
| **Sex** |  |  |  |  |  |  |  |  |  |
| Female | 1.0 (ref) |  |  |  |  | 1.0 (ref) |  | 1.0 (ref) |  |
| Male | 0.89 (0.71-1.12) | 0.321 |  |  |  | 1.52 (1.22-1.90) | <0.001 | 1.79 (1.36-2.35) | <0.001 |
| **Age (years)** | 1.03 (1.02-1.04) | <0.001 | 1.02 (1.01-1.03) | 0.028 |  | 1.04 (1.03-1.05) | <0.001 | 1.02 (1.01-1.03) | 0.003 |
| **Country of birth** |  |  |  |  |  |  |  |  |  |
| Nordic | 1.0 (ref) |  | 1.0 (ref) |  |  | 1.0 (ref) |  |  |  |
| Non-Nordic | 0.72 (0.55-0.95) | 0.019 | 0.80 (0.58-1.12) | 0.804 |  | 0.88 (0.69-1.12) | 0.880 |  |  |
| **Household income** |  |  |  |  |  |  |  |  |  |
| High | 1.0 (ref) |  | 1.0 (ref) |  |  | 1.0 (ref) |  | 1.0 (ref) |  |
| Medium | 1.00 (0.74-1.35) | 1.000 | 0.96 (0.68-1.35) | 0.808 |  | 1.23 (0.93-1.63) | 0.149 | 1.02 (0.74-1.41) | 0.906 |
| Low | 1.37 (1.08-1.83) | 0.038 | 1.47 (1.04-2.07) | 0.030 |  | 1.52 (1.15-2.00) | 0.003 | 1.54 (1.11-2.14) | 0.011 |
| **Etiology** |  |  |  |  |  |  |  |  |  |
| Viral hepatitis | 1.0 (ref) |  | 1.0 (ref) |  |  | 1.0 (ref) |  | 1.0 (ref) |  |
| Viral hepatitis + ALD | 1.29 (0.96-1.73) | 0.092 | 1.23 (0.86-1.75) | 0.259 |  | 0.73 (0.55-0.96) | 0.022 | 0.77 (0.56-1.05) | 0.095 |
| ALD | 2.21 (1.64-3.00) | <0.001 | 1.72 (1.20-2.46) | 0.003 |  | 1.48 (1.12-1.94) | 0.005 | 1.15 (0.84-1.59) | 0.379 |
| NAFLD | 2.47 (1.73-3.54) | <0.001 | 2.05 (1.31-3.21) | 0.002 |  | 3.01 (2.21-4.11) | <0.001 | 3.18 (2.12-4.77) | <0.001 |
| Other | 2.10 (1.45-2.99) | <0.001 | 1.98 (1.31-3.99) | 0.001 |  | 0.70 (0.49-1.01) | 0.055 | 0.72 (0.47-1.10) | 0.126 |
| Cryptogenic | 1.75 (1.07-2.88) | 0.026 | 1.71 (0.94-3.10) | 0.078 |  | 4.47 (3.03-6.59) | <0.001 | 3.12 (1.96-4.96) | <0.001 |
| **Decompensation**^a^ | 4.43 (3.57-5.51) | <0.001 | 4.27 (3.40-5.35) | <0.001 |  | 1.91 (1.56-2.33) | <0.001 | 2.32 (1.84-2.92) | <0.001 |
| **N of visits NPR**^b^ | 1.01 (0.99-1.02) | 0.163 | 0.99 (0.98-1.01) | 0.652 |  | 0.85 (0.83-0.88) | <0.001 | 0.84 (0.81-0.87) | <0.001 |
| **Comorbidity** |  |  |  |  |  |  |  |  |  |
| Arterial hypertension | 1.15 (0.95-1.40) | 0.159 | 0.99 (0.78-1.26) | 0.931 |  | 1.13 (0.94-1.35) | 0.199 | 0.99 (0.78-1.24) | 0.899 |
| Type 2 diabetes | 1.36 (1.12-1.66) | 0.002 | 1.07 (0.83-1.38) | 0.615 |  | 1.15 (0.95-1.38) | 0.145 | 0.84 (0.64-1.09) | 0.183 |
| CAD | 1.86 (1.40-2.48) | <0.001 | 1.58 (1.13-2.22) | 0.008 |  | 1.88 (1.44-2.46) | <0.001 | 1.63 (1.17-2.27) | 0.004 |
| ALD: Alcoholic liver disease; CAD: Coronary artery disease; CI: Confidence interval; HCC: Hepatocellular carcinoma; NAFLD: Non-alcoholic fatty liver disease; NPR: National Patient Registry; OR: Odds ratio. Nordic country of birth: Sweden, Denmark, Finland, Iceland and Norway. Household income is defined as disposable income per household per consumption unit. The cohort comprised 2670 HCC patients. Some 901 patients with recognized cirrhosis were diagnosed under surveillance: 736 patients with recognized cirrhosis were diagnosed without surveillance, and 1033 patients had unrecognized cirrhosis at the time of HCC diagnosis. Results from univariable and multivariable logistic regression models. The multivariable model 1 included all shown variables, was statistically significant compared to the null model, chi-square (14) = 257.907, p<0.001; and correctly classified 69% of cases. The multivariable model 2 included all shown variables, was statistically significant compared to the null model, chi-square (14) = 339.386, p<0.001; and correctly classified 71% of cases. Statistically significant estimates from multivariable models were highlighted. ^a^ Decompensated cirrhosis is defined as ascites or encephalopathy or bilirubin ≥52 μmol/L or albumin <28 g/L. ^b^ Registered within 365 days before HCC diagnosis. | | | | | | | | | |

| **Table S5.** Factors associated with the likelihood of having unrecognized liver cirrhosis before being diagnosed with HCC compared to patients with recognized cirrhosis diagnosed with HCC without surveillance | | | | |
| --- | --- | --- | --- | --- |
|  | **Univariable** | | **Multivariable** | |
|  | OR (95% CI) | P-value | aOR (95% CI) | P-value |
| **Sex** |  |  |  |  |
| Female | 1.0 (ref) |  | 1.0 (ref) |  |
| Male | 1.71 (1.36-2.15) | <0.001 | 1.83 (1.38-2.43) | <0.001 |
| **Age (years)** | 1.02 (1.01-1.03) | 0.001 | 1.00 (0.99-1.02) | 0.275 |
| **Country of birth** |  |  |  |  |
| Nordic | 1.0 (ref) |  | 1.0 (ref) |  |
| Non-Nordic | 1.22 (0.93-1.59) | 0.150 | 1.06 (0.76-1.48) | 0.725 |
| **Household income** |  |  |  |  |
| High | 1.0 (ref) |  | 1.0 (ref) |  |
| Medium | 1.11 (0.83-1.50) | 0.489 | 1.20 (0.84-1.71) | 0.318 |
| Low | 1.23 (0.91-1.67) | 0.187 | 1.10 (0.78-1.57) | 0.584 |
| **Etiology** |  |  |  |  |
| Viral hepatitis | 1.0 (ref) |  | 1.0 (ref) |  |
| Viral hepatitis + ALD | 0.56 (0.41-0.77) | <0.001 | 0.63 (0.43-0.92) | 0.018 |
| ALD | 0.66 (0.49-0.90) | 0.007 | 0.59 (0.41-0.85) | 0.005 |
| NAFLD | 1.22 (0.88-1.68) | 0.231 | 1.12 (0.72-1.74) | 0.603 |
| Other | 0.33 (0.23-0.49) | <0.001 | 0.35 (0.22-0.56) | <0.001 |
| Cryptogenic | 2.54 (1.66-3.89) | <0.001 | 1.87 (1.12-3.13) | 0.017 |
| **Decompensation**^a^ | 0.43 (0.35-0.53) | <0.001 | 0.47 (0.38-0.59) | <0.001 |
| **N of visits NPR**^b^ | 0.83 (0.81-0.86) | 0.163 | 0.84 (082-0.87) | <0.001 |
| **Comorbidity** |  |  |  |  |
| Arterial hypertension | 0.98 (0.81-1.18) | 0.811 |  |  |
| Type 2 diabetes | 0.84 (0.70-1.01) | 0.077 | 0.95 (0.73-1.25) | 0.719 |
| CAD | 1.01 (0.79-1.29) | 0.936 |  |  |
| ALD: Alcoholic liver disease; CAD: Coronary artery disease; CI: Confidence interval; HCC: Hepatocellular carcinoma; NAFLD: Non-alcoholic fatty liver disease; NPR: National Patient Registry; OR: Odds ratio. Nordic country of birth: Sweden, Denmark, Finland, Iceland and Norway. Household income is defined as disposable income per household per consumption unit. The cohort comprised 2670 HCC patients. Some 901 patients with recognized cirrhosis were diagnosed under surveillance: 736 patients with recognized cirrhosis were diagnosed without surveillance and 1033 patients had unrecognized cirrhosis at the time of HCC diagnosis. Results from univariable and multivariable logistic regression models. The multivariable model 1 included all shown variables, was statistically significant compared to the null model, chi-square (13) = 303.126, p<0.001; and correctly classified 67% of cases. Statistically significant estimates from the multivariable model were highlighted.  ^a^ Decompensated cirrhosis is defined as ascites or encephalopathy or bilirubin ≥52 μmol/L or albumin <28 g/L. ^b^ Registered within 365 days before HCC diagnosis. | | | | |

| **Table S6.** Different factors and their association with the likelihood of being diagnosed with HCC under non-surveillance | | | | |
| --- | --- | --- | --- | --- |
|  | **Univariable** | | **Multivariable** | |
|  | OR (95% CI) | P-value | OR (95% CI) | )-value |
| **Sex** |  |  |  |  |
| Female | 1.0 (ref) |  | 1.0 (ref) |  |
| Male | 1.20 (0.99-1.45) | 0.067 | 1.44 (1.15-1.81) | 0.001 |
| **Age (years)** | 1.04 (1.03-1.5) | <0.001 | 1.02 (1.01-1.03) | 0.003 |
| **Country of birth** |  |  |  |  |
| Nordic | 1.0 (ref) |  | 1.0 (ref) |  |
| Non-Nordic | 0.81 (0.66-1.00) | 0.059 | 0.81 (0.63-1.05) | 0.116 |
| **Household income** |  |  |  |  |
| High | 1.0 (ref) |  | 1.0 (ref) |  |
| Medium | 1.13 (0.88-1.44) | 0.343 | 1.02 (0.78-1.34) | 0.901 |
| Low | 1.45 (1.14-1.85) | 0.003 | 1.57 (1.19-2.09) | 0.001 |
| **Etiology** |  |  |  |  |
| Viral hepatitis | 1.0 (ref) |  | 1.0 (ref) |  |
| Viral hepatitis + ALD | 0.93 (0.74-1.18) | 0.572 | 0.84 (0.64-1.11) | 0.219 |
| ALD | 1.75 (1.37-2.23) | <0.001 | 1.30 (0.98-1.73) | 0.068 |
| NAFLD | 2.81 (2.10-3.76) | <0.001 | 2.46 (1.72-3.53) | <0.001 |
| Other | 1.22 (0.91-1.64) | 0.191 | 1.18 (0.84-1.67) | 0.341 |
| Cryptogenic | 3.47 (2.39-5.03) | <0.001 | 2.75 (1.78-4.26) | <0.001 |
| **Decompensation**^a^ | 2.73 (2.27-3.27) | <0.001 | 2.98 (2.46-3.63) | <0.001 |
| **N of visits NPR**^b^ | 0.97 (0.94-0.98) | <0.001 | 0.95 (0.93-0.97) | <0.001 |
| **Comorbidity** |  |  |  |  |
| Arterial hypertension | 1.14 (0.97-1.34) | 0.121 | 0.98 (0.80-1.19) | 0.826 |
| Type 2 diabetes | 1.23 (1.05-1.45) | 0.013 | 0.94 (0.76-1.17) | 0.596 |
| CAD | 1.88 (1.47-2.40) | <0.001 | 1.55 (1.17-2.07) | 0.002 |
| ALD: Alcoholic liver disease; CAD: Coronary artery disease; CI: Confidence interval; HCC: Hepatocellular carcinoma; NAFLD: Non-alcoholic fatty liver disease; NPR: National Patient Registry; OR: Odds ratio. Nordic country of birth: Sweden, Denmark, Finland, Iceland and Norway. Household income is defined as disposable income per household per consumption unit. Results from univariable and multivariable logistic regression models. The multivariable model, including all variables in this table, was statistically significant compared to the null model: chi-square (15) = 318.831, p<0.001. The model correctly classified 69% of cases. ^a^ Decompensated cirrhosis is defined as ascites or encephalopathy or bilirubin ≥52 μmol/L or albumin <28 g/L. ^b^ Registered within 365 days before HCC diagnosis. | | | | |

| **Table S7.** Survival probabilities of patients with liver cirrhosis diagnosed with HCC in Sweden between 2012 and 2018 | | | | | | |
| --- | --- | --- | --- | --- | --- | --- |
|  | **N** | **Deaths** | **1-year survival probability (95% CI)** | **5-year survival probability (95% CI)** | **Mean survival in years**  **(95% CI)** | **Median survival in years**  **(95% CI)** |
| **Overall** | 2670 | 1974 | 0.58 (0.56-0.60) | 0.24 (0.22-0.26) | 2.99 (2.87-3.13) | 1.48 (1.36-1.60) |
| Male | 2082 | 1548 | 0.58 (0.56-0.60) | 0.24 (0.22-0.26) | 2.99 (2.84-3.13) | 1.50 (1.36-1.63) |
| Female | 588 | 426 | 0.58 (0.54-0.62) | 0.25 (0.21-0.29) | 3.04 (2.75-3.32) | 1.44 (1.18-1.70) |
| **Country of birth** |  |  |  |  |  |  |
| Nordic | 2243 | 1698 | 0.58 (0.56-0.60) | 0.22 (0.20-0.24) | 2.86 (2.72-2.99) | 1.46 (1.33-1.58) |
| Non-Nordic | 427 | 276 | 0.61 (0.56-0.65) | 0.34 (0.30-0.39) | 3.71 (3.35-4.07) | 1.72 (1.18-2.25) |
| **Age groups (years)** |  |  |  |  |  |  |
| 18-49 | 92 | 47 | 0.67 (0.57-0.76) | 0.48 (0.38-0.58) | 4.78 (3.92-5.59) | - |
| 50-59 | 493 | 328 | 0.63 (0.58-0.67) | 0.35 (0.30-0.39) | 3.73 (3.40-4.06) | 1.79 (1.52-2.42) |
| 60-69 | 988 | 688 | 0.62 (0.59-0.65) | 0.28 (0.25-0.31) | 3.31 (3.09-3.54) | 1.72 (1.46-1.99) |
| 70-79 | 847 | 683 | 0.56 (0.53-0.59) | 0.16 (0.13-0.19) | 2.36 (2.17-2.55) | 1.34 (1.17-1.52) |
| 80+ | 250 | 228 | 0.37 (0.31-0.43) | 0.04 (0.02-0.08) | 1.29 (1.06-1.52) | 0.60 (0.43-0.77) |
| **Household income** |  |  |  |  |  |  |
| High | 361 | 229 | 0.68 (0.63-0.72) | 0.35 (0.29-0.40) | 3.89 (3.50-4.28) | 2.48 (1.79-3.17) |
| Medium | 1053 | 764 | 0.61 (0.58-0.63) | 0.26 (0.23-0.28) | 3.14 (2.92-3.35) | 1.58 (1.38-1.78) |
| Low | 1256 | 981 | 0.54 (0.51-0.56) | 0.20 (0.17-0.22) | 2.61 (2.43-2.79) | 1.20 (1.03-1.37) |
| **Liver cirrhosis** |  |  |  |  |  |  |
| Recognized (surveillance) | 901 | 489 | 0.83 (0.80-0.85) | 0.43 (0.40-0.47) | 4.75 (4.50-5.00) | 3.79 (3.19-4.39) |
| Recognized (no surveillance) | 736 | 614 | 0.44 (0.40-0.48) | 0.16 (0.13-0.19) | 2.14 (1.92-2.35) | 0.76 (0.63-0.89) |
| Unrecognized | 1033 | 871 | 0.47 (0.44-0.50) | 0.13 (0.11-0.16) | 2.08 (1.91-2.25) | 0.89 (0.78-1.01) |
| **Etiology** |  |  |  |  |  |  |
| Viral hepatitis | 550 | 363 | 0.61 (0.57-0.66) | 0.32 (0.28-0.36) | 3.72 (3.40-4.03) | 1.86 (1.39-2.33) |
| Viral hepatitis and alcohol | 586 | 402 | 0.64 (0.59-0.68) | 0.31 (0.27-0.35) | 3.48 (3.19-3.78) | 1.95 (1.57-2.33) |
| Alcohol | 595 | 457 | 0.62 (0.58-0.66) | 0.22 (0.18-0.25) | 2.78 (2.52-3.04) | 1.62 (1.43-1.81) |
| NAFLD | 420 | 335 | 0.50 (0.45-0.55) | 0.16 (0.12-0.20) | 2.33 (2.03-2.63) | 1.02 (0.74-1.30) |
| Other | 279 | 205 | 0.58 (0.52-0.64) | 0.26 (0.21-0.32) | 2.95 (2.56-3.34) | 1.50 (1.05-1.94) |
| Cryptogenic | 240 | 212 | 0.41 (0.35-0.47) | 0.09 (0.05-0.13) | 1.68 (1.38-1.99) | 0.65 (0.45-0.85) |
| **Comorbidity** |  |  |  |  |  |  |
| Arterial hypertension | 1490 | 1092 | 0.62 (0.59-0.64) | 0.25 (0.22-0.27) | 3.12 (2.95-3.30) | 1.70 (1.51-1.90) |
| Type 2 diabetes | 1090 | 814 | 0.58 (0.55-0.61) | 0.23 (0.20-0.26) | 2.90 (2.70-3.11) | 1.48 (1.28-1.68) |
| Coronary artery disease | 407 | 341 | 0.53 (0.48-0.58) | 0.15 (0.11-0.19) | 2.19 (1.93-2.47) | 1.11 (0.89-1.33) |
| **HCC diagnostic pathway** |  |  |  |  |  |  |
| Surveillance | 901 | 489 | 0.83 (0.80-0.85) | 0.43 (0.40-0.47) | 4.75 (4.50-5.00) | 3.79 (3.19-4.39) |
| Clinical symptoms | 1384 | 1189 | 0.43 (0.39-0.45) | 0.13 (0.11-0.15) | 1.91 (1.77-2.06) | 0.70 (0.61-0.79) |
| Incidental | 372 | 283 | 0.56 (0.51-0.61) | 0.16 (0.12-0.21) | 2.47 (2.15-2.79) | 1.27 (1.05-1.50) |
| **BCLC** |  |  |  |  |  |  |
| 0-A | 795 | 318 | 0.94 (0.92-0.95) | 0.60 (0.56-0.63) | 5.97 (5.73-6.22) | 6.85 (5.78-7.92) |
| B-D | 1818 | 1615 | 0.42 (0.39-0.44) | 0.09 (0.08-0.10) | 1.66 (1.55-1.77) | 0.72 (0.66-0.79) |
| **Treatment** |  |  |  |  |  |  |
| Transplantation | 225 | 42 | 0.98 (0.95-0.99) | 0.84 (0.78-0.88) | 7.73 (7.39-8.07) | - |
| Resection | 322 | 133 | 0.91 (0.87-0.93) | 0.56 (0.50-0.62) | 5.75 (5.35-6.15) | 6.32 (4.55-8.09) |
| Ablation | 456 | 245 | 0.92 (0.89-0.94) | 0.42 (0.37-0.47) | 4.71 (4.38-5.03) | 4.01 (3.67-4.53) |
| Palliative | 572 | 510 | 0.63 (0.59-0.67) | 0.06 (0.04-0.09) | 1.99 (1.83-2.17) | 1.42 (1.19-1.56) |
| Best supportive care | 1095 | 1044 | 0.24 (0.21-0.26) | 0.04 (0.00-0.03) | 0.94 (0.84-1.05) | 0.34 (0.30-0.38) |
| BCLC: Barcelona Clinic Liver Cancer staging system; HCC = Hepatocellular carcinoma; Household income = Disposable income per household per consumption unit; NAFLD: Non-alcoholic fatty liver disease. Variables registered at the time of HCC diagnosis if not otherwise stated. No missing values if otherwise noted. Nordic country of birth: Sweden, Denmark, Finland, Iceland and Norway. Household income refers to a person’s disposable income per consumption unit. Disposable income is the sum of all taxable and tax-free income minus taxes and negative transfers. The income includes gains/losses, that is, the gain/loss arising from a sale (realization) of assets, for example, stocks, mutual funds or real estate. To make comparisons of, for example, disposable income and economical purchasing power between different types of households, a weight system is used where consumption is related to the composition of the household. Disposable income is divided by the weight of consumption of the household. The scale is determined by Statistics Sweden and is based on, among other things, budget calculations carried out by the Swedish Consumer Agency and the basis for assessing a basic consumption that can be calculated for different household types. The consumption unit scale used by Statistics Sweden is: single or living alone 1.0; cohabiting couple 1.51; additional adult 0.6; first child 0-19 years 0.52; second and subsequent children 0-19 years 0.42. | | | | | | |

| **Table S8.** Univariable and multivariable estimates for overall mortality in 2139 patients with cirrhosis diagnosed with HCC in Sweden between 2012 and 2018 | | | | | | |
| --- | --- | --- | --- | --- | --- | --- |
|  | **Univariable** | | **Multivariable 1** | | **Multivariable 2** | |
|  | HR (95% CI) | p-value | aHR (95% CI) | p-value | aHR (95% CI) | p-value |
| **Sex** |  |  |  |  |  |  |
| Female | 1.0 (ref) |  |  |  |  |  |
| Male | 1.05 (0.93-1.20) | 0.429 |  |  |  |  |
| **Age (years)** | 1.03 (1.02-1.04) | <0.001 | 1.02 (1.01-1.03) | <0.001 | 1.01 (1.00-1.02) | 0.002 |
| **Country of birth** |  |  |  |  |  |  |
| Nordic | 1.0 (ref) |  | 1.0 (ref) |  | 1.0 (ref) |  |
| Non-Nordic | 0.81 (0.70-093) | 0.003 | 0.75 (0.64-0.88) | <0.001 | 0.91 (0.78-1.07) | 0.260 |
| **Household income** |  |  |  |  |  |  |
| High | 1.0 (ref) |  | 1.0 (ref) |  | 1.0 (ref) |  |
| Medium | 1.32 (1.12-1.56) | <0.001 | 1.18 (0.99-1.41) | 0.058 | 1.29 (1.08-1.53) | 0.004 |
| Low | 1.58 (1.34-1.86) | <0.001 | 1.40 (1.17-1.67) | <0.001 | 1.42 (1.19-1.69) | <0.001 |
| **Liver cirrhosis** |  |  |  |  |  |  |
| Recognized (composite) | 1.0 (ref) |  | 1.0 (ref) |  | 1.0 (ref) |  |
| Unrecognized | 2.53 (2.29-2.81) | <0.001 | 2.15 (1.92-2.41) | <0.001 | 1.75 (1.57-1.96) | <0.001 |
| **Etiology** |  |  |  |  |  |  |
| Viral hepatitis | 1.0 (ref) |  | 1.0 (ref) |  | 1.0 (ref) |  |
| Viral hepatitis + ALD | 1.02 (0.87-1.20) | 0.833 | 0.94 (0.78-1.12) | 0.462 | 0.99 (0.83-1.18) | 0.917 |
| ALD | 1.27 (1.09-1.49) | 0.003 | 0.96 (0.80-1.15) | 0.654 | 1.12 (0.95-1.33) | 0.192 |
| NAFLD | 1.67 (1.41-1.97) | <0.001 | 1.21 (0.98-1.49) | 0.085 | 1.44 (1.17-1.77) | <0.001 |
| Other | 1.14 (0.93-1.39) | 0.225 | 1.09 (0.87-1.37) | 0.446 | 1.09 (0.88-1.35) | 0.450 |
| Cryptogenic | 2.34 (1.94-2.81) | <0.001 | 1.44 (1.16-1.79) | <0.001 | 1.36 (1.11-1.68) | 0.003 |
| **Decompensation**^a^ | 2.25 (2.01-2.52) | <0.001 | 2.34 (2.08-2.62) | <0.001 | Excluded |  |
| **Comorbidity** |  |  |  |  |  |  |
| Arterial hypertension | 0.88 (0.80-0.98) | 0.015 | 0.77 (0.68-0.86) | <0.001 | 0.79 (0.70-0.88) | <0.001 |
| Type 2 diabetes | 1.00 (0.90-1.11) | 0.956 | 0.96 (0.84-1.10) | 0.553 | 0.91 (0.79-1.04) | 0.151 |
| CAD | 1.40 (1.23-1.61) | <0.001 | 1.23 (1.06-1.44) | 0.007 | 1.12 (0.97-1.29) | 0.120 |
| **BCLC** |  |  |  |  |  |  |
| 0-A | 1.0 (ref) |  | Excluded |  | 1.0 (ref) |  |
| B-D | 4.97 (4.36-5.66) | <0.001 | Excluded |  | 4.08 (3.56-4.67) | <0.001 |
| ALD: Alcoholic liver disease; BCLC: Barcelona Clinic Liver Cancer; CAD: Coronary artery disease; CI: Confidence interval; HCC: Hepatocellular carcinoma; HR: Hazard ratio; NAFLD: Non-alcoholic fatty liver disease. Nordic country of birth: Sweden, Denmark, Finland, Iceland and Norway. Household income is defined as disposable income per household per consumption unit. Cox regression models were used to calculate HR and adjusted HR (aHR) for death. Each patient was followed-up from the date of HCC diagnosis until the date of death, or until 31 December 2020, whichever occurred first. Recognized liver cirrhosis comprised patients diagnosed with HCC under surveillance (n = 901) and patients diagnosed under non-surveillance but who might have been surveilled before HCC diagnosis (n = 205). Unrecognized liver cirrhosis comprised 1033 patients diagnosed with liver cirrhosis at the time of or shortly after HCC diagnosis. ^a^ Decompensated cirrhosis is defined as ascites or encephalopathy or bilirubin ≥52 μmol/L or albumin <28 g/L | | | | | | |

| National Patient Register  SweLiv  Hospital discharge letters  Outpatient speciality care letters  Cause of Death Register  1997  2001  2012  2018  2019  Year  Prescribed Drug Register  2005 |
| --- |
| **Fig. S1.** Swedish nationwide registers: |
| - The National Patient Register (NPR) contains data from hospital discharge letters (1997-2019) and outpatient specialty care letters (2001-2019). The NPR was first established in 1964 and has had national coverage since 1987. This register does not include data from primary care visits. |
| - The Prescribed Drug Register has data available since July 2005. The register includes data from prescribed drugs dispensed at any Swedish pharmacy but does not include over-the-counter medications. |
| - SweLiv is the Swedish quality register for cancers found in the liver, the gallbladder, and the bile ducts. SweLiv was established in 2008 and currently includes>95% of all known HCC cases in Sweden. SweLiv consists of four modules: 1) diagnosis, staging, and treatment recommendations, 2) interventions, 3) surgical complications and pathology, and 4) follow-up. |
| - The Cause of Death Register was established in 1911 and is currently >99% complete. |
| 175  630  1637  858  2072  Underreported SweLiv (598)  t_0_ - 30 days  t_0_ = time of HCC diagnosis  1214  423  175  858  t_0_ + 180 days  1997  455  Undercoded NPR (403)  403  SweLiv  NPR  Recognized liver cirrhosis  Unrecognized liver cirrhosis |
| **Fig. S2.** Classification of recognized and unrecognized cirrhosis in 2670 patients diagnosed with hepatocellular carcinoma (HCC) in Sweden between 2012 and 2018. Cirrhosis was defined as having at least one ICD-10-SE cirrhosis-related code registered in the National Patient Register (NPR) from 1997 and up to 180 days after HCC diagnosis or as having cirrhosis registered in the Swedish quality register for cancers found in the liver, gallbladder, and bile ducts (SweLiv). Patients with at least one International Classification of Diseases 10^th^ Revision – Swedish Edition (ICD-10-SE) cirrhosis-related code registered in the NPR from 1997 and up to 30 days before HCC diagnosis were classified as recognized cirrhosis or otherwise as unrecognized cirrhosis. |

| Transplant candidate?  Terminal stage  Single or ≤ 3 nodules ≤ 3 cm  CP ≤ 7^a^  ECOG PS 0–1 (2^b^)  M0, N0  Multinodular  CP ≤ 7^a^  ECOG PS 0-1 (2^b^)  M0, N0  CP ≤ 7  ECOG PS 0–1  M1, N1  CP > 7  ECOG PS ≥ 2  Comorbidity  Resection/ablationcandidate?  Resection/  ablation  Transplant  TACE candidate?  TACE  Systemic chemotherapy  Best supportive care  UCSF  Criteria  Early stage  Intermediate stage  Advanced stage  Transplant candidate?  Transplant candidate?  Resection/ablation candidate?  Transplant  Transplant  Resection/  ablation  Yes  Downsizing  No  Yes  No  Yes  Yes  No  Yes  No  No  Yes  Yes  No  Yes  Treatments with curative intent  Non-curative treatments  Symptomatic therapy  TACE candidate? |
| --- |
| **Fig. S3.** Swedish treatment algorithm for hepatocellular carcinoma. CP = Child-Pugh score; ECOG PS= Eastern Cooperative Oncology Group performance status; M = Distant metastasis; N = Regional lymph node metastasis; TACE = Transarterial chemoembolization; UCSF = University of California San Francisco. ^a^ CP non-relevant for liver transplant candidates. ^b^ Some patients with ECOG PS = 2 might become candidates for treatment with curative intent after individual evaluation at multidisciplinary conferences.  This figure has been reproduced and adapted after Vaz *et al.* (Targeting population groups with a heavier burden of hepatocellular carcinoma incidence: A nationwide descriptive epidemiological study in Sweden. Int J Cancer. 2022 Jul 15;151(2):229-239. Attribution-NonCommercial 4.0 International [CC BY-NC 4.0]). |

|  |
| --- |
| **Fig. S4.** Percentage of visits registered in the National Patient Register within 365 days before hepatocellular carcinoma (HCC) diagnosis for 735 patients with unrecognized cirrhosis, later diagnosed with HCC in Sweden between 2012 and 2018. Some 3073 visits were registered, of which 75% were outpatient visits. |
